# Therapeutic efficacy of voltage-gated sodium channel inhibitors in epilepsy

**DOI:** 10.1186/s42494-023-00127-2

**Published:** 2023-06-28

**Authors:** John Agbo, Zainab G. Ibrahim, Shehu Y. Magaji, Yahkub Babatunde Mutalub, Philemon Paul Mshelia, Daniel H. Mhya

**Affiliations:** 1 Department of Clinical Pharmacology and Therapeutics, Faculty of Basic Clinical Sciences, College of Medical Sciences, Abubakar Tafawa Balewa University, Bauchi, 740272 Nigeria; 2 Department of Physiology, Faculty of Basic Medical Science, College of Medical Sciences, Abubakar Tafawa Balewa University, Bauchi, 740272 Nigeria; 3 Department of Medical Biochemistry, Faculty of Basic Medical Science, College of Medical Sciences, Abubakar Tafawa Balewa University, Bauchi, 740272 Nigeria

**Keywords:** Epilepsy, Voltage-gated sodium channels, Idiopathic epilepsy, Acquired epilepsy, Anti-seizure medications, Pharmacoresistant epilepsy

## Abstract

Epilepsy is a neurological disease characterized by excessive and abnormal hyper-synchrony of electrical discharges of the brain and a predisposition to generate epileptic seizures resulting in a broad spectrum of neurobiological insults, imposing psychological, cognitive, social and also economic burdens to the sufferer. Voltage-gated sodium channels (VGSCs) are essential for the generation and propagation of action potentials throughout the central nervous system. Dysfunction of these channels has been implicated in the pathogenesis of epilepsy. VGSC inhibitors have been demonstrated to act as anticonvulsants to suppress the abnormal neuronal firing underlying epileptic seizures, and are used for the management and treatment of both genetic-idiopathic and acquired epilepsies. We discuss the forms of idiopathic and acquired epilepsies caused by VGSC mutations and the therapeutic efficacy of VGSC blockers in idiopathic, acquired and pharmacoresistant forms of epilepsy in this review. We conclude that there is a need for better alternative therapies that can be used alone or in combination with VGSC inhibitors in the management of epilepsies. The current anti-seizure medications (ASMs) especially for pharmacoresistant epilepsies and some other types of epilepsy have not yielded expected therapeutic efficacy partly because they do not show subtype-selectivity in blocking sodium channels while also bringing side effects. Therefore, there is a need to develop novel drug cocktails with enhanced selectivity for specific VGSC isoforms, to achieve better treatment of pharmacoresistant epilepsies and other types of epileptic seizures.

## Background

Epilepsy is characterized by recurrent seizures due to aberrant excessive discharges of cortical neurons [1]. Epilepsy is a chronic brain disease that affects about 70 million people all over the world [2–5]. Nearly 80% of individuals with epilepsy live in low-and middle-income countries with limited resources and high poverty rate (sub-Saharan Africa, Latin America, southeast Asia), where the rate of new cases is more than two-fold higher than that in developed countries [6]. Epilepsies are divided into two categories [1] genetic epilepsy with no known structural, gross neuroanatomic, or neuropathologic abnormalities or predisposing factors but being primarily due to underlying genetic mutations [7], and [2] symptomatic acquired epilepsy, which is associated with gross anatomic and pathologic abnormalities, resulting from structural or metabolic perturbations in the brain [8]. Seizures can have multifactorial mechanisms, and they often appear so diverse that one would suspect that there is no common connotation. However, it is commonly believed that seizures arise when the homeostatic mechanisms are disrupted, causing an imbalance between excitation and inhibition. Normally, there are checkpoints that keep neurons from excessive action potential (AP) discharging, and also mechanisms that facilitate neuronal firing so that the nervous system can function normally. Homeostatic disruption of the checkpoints or promotion of the mechanisms that enhance excitation can lead to seizures. Currently, there is no cure for epilepsy. VGSC inhibitors and other anti-seizure medications (ASMs) are only aimed to suppress seizures. In addition, some VGSC inhibitors such as phenytoin, carbamazepine, and lamotrigine as well as other types of ASMs are contraindicated for some forms of genetic epilepsy that are caused by mutations in the α and β subunits of sodium channels. They do so by blocking sodium currents entering the neurons. Examples of such epilepsies include Dravet syndrome (also known as severe myoclonic epilepsy in infancy, SMEI), generalized (genetic) epilepsy with febrile seizures plus (GEFS +) and benign familial neonatal infantile seizures (BFNIS). Therefore, it is imperative to enhance recognition of the disease mechanisms, the molecular structure of sodium channel and physiological roles of VGSC subtypes, in order to develop new drugs that can modulate sodium currents and change the inactivation characteristics.

## Physiological and molecular architecture of VGSCs

VGSCs are of great significance to the initiation of APs in neurons and other excitable cells [9], and their dysfunction causes epilepsy, inherited diseases of hyperexcitability and related channelopathies. VGSCs function by transiently increasing the membrane permeability to sodium ions during membrane depolarization. At resting states these membranes are usually closed. The depolarization of the membrane induces a conformational change of the α subunit through movements of the voltage-sensing domains, prompting the opening of the sodium-selective channel pore. Voltage-gated sodium channels open very fast within 1–2 ms, which is required for repetitive AP firing in neural circuits and for control of excitability in nerve and muscle cells [10]. Within a few milliseconds, the channels rapidly shift to a nonconducting inactivated state, mediated by the triad isoleucine-phenylalanine-methionine (IFM) motif of the α subunit [11]. Conversely, slow channel inactivation ensues as a result of long depolarization of nerve and muscle fibers mediated by inward trains of currents, with concomitant repetitive neuronal firing for a period of seconds due to the long-term changes in the resting membrane potential. Usually, VGSCs are highly dynamic transient channels that are inactivated and closed within milliseconds. However, under certain conditions, deficits or attenuation of fast inactivation can greatly enhance the amplitude of persistent sodium currents, resulting in bursts of APs as seen in paroxysmal epileptic seizures, pain and even cardiac arrhythmia.

Since the groundbreaking chemical characterization of VGSCs by Beneski and Catterall [12], efforts have been made to unravel structures and physiological roles of sodium channels. The mammalian VGSC is a complicated composed of a large, pore-forming α subunit of 260 kDa and one or two smaller β (auxiliary) subunits of 34–40 kDa. The ion-conducting pore, also known as the pore-forming domain, is contained within the α subunit. The α subunit, as the dominant subunit of VGSC, is responsible for normal electrophysiological function and mediates the fundamental physiologic properties of VGSC, including rapid inactivation. The sodium channel α subunit family have nine members, encoded by nine genes and expressed in different excitable tissues [13]. Mutations in these channels can result in genetic epilepsy [14] and other channelopathies [15]. The tenth VGSC is involved in salt-sensing and is not voltage-gated [16]. The 10 homologous α subunits of VGSCs found in humans are designated Nav1.1–Nav1.9 and NaX, encoded by ten different genes (*SCN1A-SCN11A, SCN6A* and *SCN7A* represent the same gene). Nav1.1, Nav1.2, Nav1.3 and Nav1.6 (*SCN1A, SCN2A, SCN3A, SCN8A*) are expressed in the central nervous system (CNS). Nav1.7, Nav1.8 and Nav1.9 (*SCN9A, SCN10A, SCN11A*) are expressed in the peripheral nervous system. Nav1.4 (*SCN4A*) is the primary sodium channel in skeletal muscle, while Nav1.5 (*SCN5A*) is the canonical subtype in the heart [17]. The tenth isoform NaX (*SCN7A*) is voltage-insensitive and is considered atypical as it contains key distinguishing features in DI/III/IV S4 of voltage-sensor domains (VSDs) and DIII-IV linker sequence. Therefore, NaX is classified as a different type of Nav [18]. Apart from the primary tissue of expression, most of the sodium channels have expressions in other tissues.

The α subunit is the core subunit of VGSC, and is composed of three parts: (1) four highly homologous transmembrane domains designated as DI-DIV, with each domain harboring six nearly identical transmembrane segments with sequence homology greater than 50% [18]; (2) three intracellular loops (2 long loops, L1 and L2, and 1 short loop, L3); and (3) the N- and C-termini (NT and CT). The CT of NaV1.7 is reported to be involved in orchestrating the process of fast inactivation, which forms two electrostatic bridges with gating charges in VSD4 (switch 1) and DIII-DIV linker (switch 2), respectively [19]. Each domain comprises six water-filled α-helical transmembrane segments named S1-S6. Transmembrane segments S1-S4 of the sodium voltage-gated channel α subunits from each of the four domains form the voltage-sensing domain, an essential structure that function in the modulation of channel opening upon depolarization of the membrane. The VSD can move easily due to the presence of positively charged amino acid residues arginine and lysine at S4, which are exceedingly sensitive to the change of the membrane potential. For that reason, segment S4 is called the voltage sensor of the VGSC. The intracellular loop L3 connecting the homologous domains DIII-DIV constitute the inactivation gate that serves as a hinged lid and folds into the intracellular mouth of the pore during fast inactivation [20]. Defective fast inactivation of this hinged lid or if for any reason the hinged lid is left ajar for more than the required milliseconds, trains of Na^+^ will rush in, causing excessive depolarization of the membrane and excessive neuronal firing (persistent sodium currents). Both of the CT and NT of the subunit also modulate the VGSCs, for example, the CT plays a crucial role in inactivation. Many mutations causing human diseases related to inactivation are identified in the CT of the sodium channels [21–25].

The α subunit of the VGSC is coupled to one or two β subunits called auxiliary subunits. The β subunits have unique functions independent of the α subunit: cell adhesion and intracellular/extracellular signaling [26–30].

### Roles of VGSCs in genetic and acquired epilepsies

Based on the site of origin, genetic epilepsy (also known as idiopathic epilepsy (ies)) can be either focal or generalized epileptic seizures. Although these seizures lack a known cause, they are considered to be genetically determined. Genetic epilepsies do not show lesional neuropathologic abnormality, and have normal brain imaging presentation. They are estimated to represent about 47% of all epilepsies [31]. These types of epilepsies are mostly precipitated by gene mutations encoding ion channels or their ancillary subunits. The genetic defects can be either monogenic or polygeneic, with monogenic defects accounting for a small proportion (~ 2%). A good example of monogenic etiology is the benign familial neonatal seizures (BFNS), which are the first discovered CNS channelopathy and the best known genetically determined human epilepsies. Polygenic defects with convoluted polygenic traits account for a high percentage of idiopathic genetic epilepsies (IGE). Although numerous genetic mutations have been found to cause some genetic-idiopathic epilepsies in humans [32–36], some forms of idiopathic epilepsies still have unclear causes. However, ion channel defects are widely recognized as one of the major causes of idiopathic epilepsies. Genetic-idiopathic epilepsies can be caused by dysfunctions of voltage-gated ion channels (VGICs) which are essential for AP generation and maintenance of resting membrane potentials, or by ligand-gated ion channels (LGICs) which are mainly responsible for synaptic transmission. Mutations in VGICs (Na^+^, K^+^, Cl^−^, Ca^2+^ channels) and LGICs (N-methyl-D-aspartate receptors, nicotinic acetylcholine receptors [nAChRs], γ-Aminobutyric acid sub-type A [GABA_A_] receptors) can cause neuronal hyperexcitability through several pathogenic mechanisms. The CNS is abundantly enriched with VGICs, which are responsible for the generation, propagation, regulation of neuroexcitability and are therefore regarded as key players in the pathogenesis of epilepsy especially when the homeostatic mechanism goes awry. Idiopathic epilepsies are predominately due to the genes mutation encoding for ion channels. Although ion channel genes mutation contributes to only a small fraction (27%) of all genetic epilepsies (LGICs 10%, VGICs 17%) [37], they have received much attention from studies on genetic epilepsies and channelopathies. Understanding the role of ion channels in epilepsy can provide insight into the disease mechanisms, precision diagnosis and classification of epileptic syndromes, and promote drug design and development, validation of new drug target as well as development of pharmacotherpeutic strategies and interventions. Most of our understanding of molecular signatures of epilepsy in general came to the fore in 1995 when Steinlein and Colleagues reported for the first time that a missense mutation in the neuronal nACHR α4 subunit corresponds with autosomal-dominant nocturnal frontal lobe epilepsy (ADNFLE) [38]. Apart from nACHRs, mutations in a plethora of other genes have also been implicated in the epileptogenesis of ADNFLE, including *DEPDC5* (22q12.3), *CRH* (8q13) and *CABP4* (11q13.2). Examples of epilepsy caused by genetic mutations of ion channels include epilepsy caused by VGSC mutations. The important roles of VGSCs in neurohyperexactibility have made them potential candidates for episodic neurological disorders as seen in epileptic seizures. Usually, the VGSCs become permeable to sodium when the channels are open, and sodium ions flow into the intracellular space from the extracellular space (activated state). The opening of the channel is orchestrated by the DI–DIII S4 voltage sensors, which undergo rapid movement in response to altered electric field across the cell membrane due to depolarization, resulting in a conformational change in the protein [39]. After a few milliseconds, inactivation occurs, mediated by the IFM triplet located in the highly conserved intracellular linker connecting domains DIII and DIV. The inactivation gate plays the role of hinged seal and folds into the channel pore during fast inactivation [11]. Unfortunately, however, incomplete inactivation in some neurons either by mutations or temperature (fast and slow inactivation) have been involved in the pathogenesis of epilepsy, through generation of persistent current, a current activated at subthreshold voltages that enhances epileptic burst firing by decreasing the threshold for AP generation, sustaining repeated firing and augmenting depolarizing synaptic currents [40]. Mutations in VGSCs (such as in *SCN1A, SCN2A, SCN3A,* and *SCN8A*) can lead to defects in inactivation gating, enhancing persistent sodium currents (*I*_NaP_) and firing of neurons, resulting in epilepsy and ataxia [41].

There are two major types of genetic epilepsy associated with VGSC dysfunction or mutation, the idiopathic generalized epilepsy (IGE) and idiopathic focal epilepsy. IGE is believed to be polygenic, and encompasses a continuum of epileptic seizures like absence seizures, myoclonic seizures and generalized tonic–clonic seizures. Two well-known examples of IGE with VGSC mutation implications are Dravet syndrome (*SCN1A*) and genetic epilepsy with febrile seizure plus GEFS + (*SCN1A, SCN1B*). BFNIS is a classic example of idiopathic focal epilepsy caused by VGSC mutations. Other rare monogenic idiopathic epilepsy syndromes like BFNS and ADNFLE are not caused by mutations of VGSCs and will not be discussed further here. Although there are fewer global genetic phenotypes or syndromes caused by VGSC mutations than by other VGICs, a particular VGSC gene can harbor plenty of mutations. For example, the Na_V_1.1-encoding *SCN1A* gene, whose missense mutation causes DS, has been found with ~ 600 mutations in its sequenced coding sequences, representing 70% of cases [42]. The importance of VGSCs is not only because that they are responsible for the generation of APs but also that they harbor mutations that are responsible for the epileptogenesis of rarer genetic epileptic syndromes and many epileptic encephalopathies that are intractable and pharmacoresistant to ASMs. Most importantly, most notable ASMs exert their effects by modulating or manipulating the VGSCs. Among the genetic-idiopathic epilepsies in which VGSCs are implicated, Dravet syndrome (also known as SMEI) is a type of highly debilitating, recalcitrant and pharmacoresistant epilepsy resulted from missense mutations in the VGSC protein NaV1.1 encoded by the *SCN1A* gene [43]. Other forms of epileptic syndromes caused by mutations in VGSCs are generalized (genetic) epilepsy with febrile seizure plus + (GEFS), a milder form of epilepsy compared to Dravet syndrome, resulted from the mutations of *SCN1A* and *SCN1B* (which encodes the β1 subunit of nACHR); and BFNIS, which is caused by mutations in *SCN2A*, a gene encoding one of the α-subunits of VGSCs. Intractable childhood epilepsy generalized tonic-colonic is another type of epileptic seizure caused by VGSC mutations, which is similar to SMEI in many aspects, including pharmacoresistance, intractability, age onset, fever association and learning disability [44, 45].

Unlike genetic idiopathic epilepsies that present no structural lesions or other predisposing causes, acquired epilepsies are characterized by visible structural lesions and neuroanatomic features. Acquired epilepsies start from a particular point around the structural lesion and therefore have a focal origin of bursting. The electroencephalogram pattern and clinical presentation of acquired epilepsies depend on the particular brain region where the seizures start and spread and can range from mild, moderate to severe. Acquired epilepsies are triggered by neuropathological insults and about 50% of all epilepsies are acquired. Examples of common brain injuries or insults that trigger acquired epilepsies are traumatic brain injury, hippocampal sclerosis, tumors, stroke and status epilepticus. Although VGSC mutations are mostly implicated in genetic epilepsies, evidence shows that aberrant functions and mutations of VGSCs are involved in the pathogenic mechanism of acquired epilepsies. This is because acquired epilepsies are mostly triggered through the process of epileptogenesis, a process of transformation from a functional balance between excitation and inhibition to hyperexcitability of neurons [46]. VGSCs have been implicated in acquired epilepsies through acquired channelopathies via generation of aberrant large persistent sodium current (*I*_NaP_) as observed in genetically normal rodents with acquired epilepsies [47]. It is already known that mutations in any of the genes for Nav1.1, Nav1.2, Nav1.3 and Nav1.6 that are present in the CNS result in diverse forms of genetic epilepsies including the severe refractory epilepsy like Dravet syndrome. However, what is fascinating now is that mutations of these genes causing the elevations of *I*_NaP_ also result in acquired epilepsies through epileptic encephalopathy syndromes more lethal and severe than Dravet syndrome such as Lennox-Gastaut Syndrome (LGS) and sudden unexpected death in epilepsy (SUDEP) [48].

## Mechanisms of action of ASMs that act through VGSCs

A majority of anti-seizure agents are designed to create a balance that favor inhibition over excitation and therefore stop or prevent seizure activity [49]. Although there is no permanent cure for epilepsy, the symptomatic remission or relief from seizures by ASMs occurs through various mechanisms and interactions with different cellular targets [50, 51]. The mechanisms of ASMs can be classified into four major types: 1) modulation of VGICs such as calcium, sodium, and potassium channels; 2) potentiation of GABA-mediated inhibition through effects on GABA_A_ receptors, GABA transporter 1, GABA-synthesizing enzyme glutamic acid decarboxylase, or the GABA-metabolizing enzyme GABA transaminase; 3) direct modulation of synaptic release through effects on components of the release machinery, including synaptic vesicle protein 2A; and 4) inhibition of synaptic excitation mediated by ionotropic glutamate receptors including AMPA receptors. ASMs act through VGSCs because the flow of cations across cell membranes is mediated via VGICs. VGSC mediates the rising phase of APs, during which the channel allows increased influx of sodium ions into the cell. Enormous neuronal excitation and excessive electrical discharge result in epileptic seizures. Therefore, VGSCs have been studied as a therapeutic target for epilepsy. ASMs acting as sodium channel inhibitors stabilize sodium channels by preventing them to return to the active state and potentiating the inactive state, thereby preventing repetitive firing of axons and neuronal depolarization. ASMs such as phenytoin, carbamazepine, oxcarbazepine, zonisamide and lamotrigine, inhibit abnormal epileptiform activities by blocking the fast inactivation state of VGSCs [52]. They bind to the inactivated voltage-gated channels after depolarization and modify their permeability to sodium ions, thereby reducing inward sodium movement. This leads to an enhancement in the inactivation (or refractory) period of frequently firing neurons. ASMs can manipulate either the fast- (phenytoin, carbamazepine, Fosphenytoin, oxcarbazepine, primidone, zonisamide, and valproic acid [VPA]) or the slow-inactivation (lacosamide and eslicarbazepine) gate or state of the VGSCs.

## The role of phosphorylation of VGSCs in the pathogenesis and treatment of epilepsy

Phosphorylation is one of the most common post-translation modifications (PTMs) at the proteomic level, and together with N-glycosylation, is considered as the most abundant PTM [53]. Phosphorylation is also the most widely studied PTM in sodium channels. Although the molecular mechanisms of aberrant expression, localization, as well as function of Nav channels in the development of epilepsy is poorly understood, it is considered that it may be caused by altered PTMs. Phosphorylation modulating VGSC gating, and has been thought to be the cause of acquired insensitivity of Nav channels to anti-seizure medications in epileptic neurons. Neverthless, whether the changes of PTMs of specific Nav channels occur sharply during epileptic seizures remain unclear. Several sites of phosphorylation have been identified by proteomic profiling and mass spectrometry, although there is paucity of data on which protein kinase(s) catalyse the phosphorylation. Specifically, latest mass spectrometry-based proteomic analyses of Nav1.2 purified from rat brain [54] or present in whole mouse brain phosphoproteome fractions. Two different monoclonal antibodies one specific for Nav1.2, and one with pan-VGSC specificity, have been used in parallel immunopurification and MS analyses of rat brain VGSC phosphorylation. These studies characterized fifteen phosphosites on Nav1.2, and three on Nav1.1, making Nav1.2 the VGSC with the highest phosphosites [55, 56] have identified > 60 in vivo phosphorylation sites on brain Nav1.2, much more than those identified on any other Nav channels. Nonetheless, cAMP-dependent kinase (PKA) and protein kinase C (PKC) have long been known to phosphorylate brain VGSC [57–59]. The intracellular domains of the VGSC are targets for phosphorylation at multiple sites [60, 61]. Apart from PKA and PKC, other kinases for brain VGSC are glycogen synthase kinase 3 (GSK3) [62, 63], a kinase-anchoring protein 15 [64], Fyn tyrosine kinase [65], as well as p38 mitogen-kinase activated protein kinase [66]. Many of these phosphorylations are related to the pathogenesis of genetic and acquired epilepsies. Therefore, identifying the signal pathway of dysfunction in epilepsy might supply new targets for anti-seizure medications [67].

The electrophysiological effects of phosphorylation on VGSC are often dependent on the specific isoform. PKA and PKC phosphorylation of Nav1.2 causes defective channel trafficking to the cell surface, resulting in attenuation of Nav1.2 currents [59]. Increased phosphorylation of Nav1.2 in the ID I-II linker region is usually related to the decrease of Nav current [57, 68, 69]. As evidence shows some effects of topiramate (TPM) on AMPA/kainate receptors are affected by the phosphorylation state of the receptors, TPM may bind to the phosphorylation sites of these receptors in the inner loop, thereby modulating ionic conductance via the channels allosterically. TPM may also prevent PKA and PKC from phosphorylating the channels. This suggests the crucial role of phosphorylation in the pathogenesis of epilepsy and its manipulation to exert anti-seizure effects [70]. Phosphorylation signaling pathways such as the p38 MAPK-JNK signaling are important regulators of cellular function and may be a target for drug design and development [71, 72].

## Therapeutic effectiveness of VGSC inhibitors in genetic and acquired epilepsies

ASMs, formerly referred as anti-epileptic drugs or anticonvulsant, are the main treatment for both genetic and acquired epilepsies. To date, most of the mutations established to be related to epilepsy locate in genes encoding VGSCs. Mutations in the 9 different α isoforms of VGSC (NaV1.1-NaV1.9) are reported to cause channelopathies. Specifically, mutations of genes for NaV1.1 (*SCN1A*), NaV1.2 (*SCN2A*), NaV1.3 (*SCN3A*), NaV1.6 (*SCN8A*) and NaV1.7 (*SCN9A*) are related to both genetic and acquired epilepsies because of their abundant presence in the CNS. Seizures are precipitated by bursts of high-frequency APs, and ASMs might inhibit seizures by impeding the bursts by gradually inhibitng VGSCs. The VGSCs can transit through multiple states, and ASMs have varying affinities to the channel depending on the state [73]. All these modulations and manipulations are possible because the inactivation of VGSC is typically characterized with fast and slow components. The fast component occurs within 5–10 ms, while the slow component may take hundreds of milliseconds to initiate. Considering the central role of VGSCs in regulating neuronal excitability, many common ASMs exert their putative actions by targeting VGSC function. Therefore, blocking or inhibiting VGSCs during excessive hyperexcitation seems to be a sensible way to repress or suppress seizures. However, some epilepsies are believed to arise from specific loss of VGSCs in inhibitory neurons, leading an imbalanced excitatory-inhibitory (E-I) ratio. In such cases, an activator of VGSC could restore the channel function in inhibitory neurons. Compounds that stimulate or selectively activate NaV1.1 are new targets to achieve this goal. Given the fact that NaV1.1 expressed predominantly in inhibitory interneurons, NaV1.1 activation is assumed to enhance overall inhibition and prevent seizures potentially [74]. The main mechanism of action of these ASMs seems to be use-dependent block, that is, when the membrane potential experiences repeated reach to depolarized levels more frequently, inhibition of sodium currents is stronger, exposing novel drug-binding sites and selectively blocking of channels was only allowed when they are in the active neurons [75]. Dysfunction of many subtypes of VGSCs may lead to the development of epilepsy. Below we discuss and summarize how dysfunction of some subtypes of VGSCs leads to the pathogenesis of epileptic seizures and how biophysical manipulations of these VGSCs could be used as an approach to the treatment of genetic, acquired, Dravet syndrome, Lennox Gastaut syndrome and other pharmacoresistant epilepsies.

## Putative roles of Nav1.1 and Nav 1.2 in genetic and acquired epilepsies and epilepsy management

It is already known that VGSCs take charge of the initiation of APs in neurons, and inhibitors of sodium channels are used for treatment of epilepsy. Sodium channel activators were not considered to be therapeutically relevant due to their toxicity and side effects. However, selective activators of the NaV1.1 sodium channel might be potentially therapeutic for diseases, including epilepsy [76]. Like Nav1.2, Nav1.3, and Nav1.6, Nav1.1 also has high expression level in the CNS. It is well known that in the process of modulating GABAergic inhibitory interneuron physiology, Nav1.1 plays a vital role. Many mutations in sodium channels can cause inherited epilepsy syndromes of different severities, and among these mutations, the NaV1.1 channel encoded by the *SCN1A* gene is the most common target [77, 78]. *SCN1A*, which encodes the Nav1.1 subtype of the pore-forming α subunit of the VGSCs, has been identified with 200 epilepsy mutations [79, 80]. In fact, of all known mutations of epilepsy genes, *SCN1A* mutations are the most diversely implicated in both hereditary and acquired seizure pathogenesis [8, 81]. Most Dravet syndrome and GEFS + cases have mutations in *SCN1A,* which suggests in the context of epilepsies, this channel plays a role. NaV1.1 causes epilepsy either by gain or by loss of function of sodium channels that either increase or decrease neuronal excitability via a widespread dysfunction of network inhibition. It is hypothesized that in the context of epilepsy, Due to its ability and propensity to attenuate Nav1.1 sodium current and resulting in reducing the excitability of inhibitory neurons, Nav1.1 mutations, regardless of being missense or nonsense, gain-of-function or loss-of-function, and their association with GEFS + or SMEI, all stem from it. In fact, reflecting upon individual genetic differences, a spectrum of diseases from GEFS + to SMEI, all reflect a certain extent of Nav1.1 attenuation, whether it be partial or complete. Several lines of evidence suggest that loss-of-function mutations in VSGCs cause epileptic disorders [82, 83]. SMEI (or Dravet's Syndrome) is caused by complete loss-of-function mutations in NaV1.1, which is a severe and intractable epilepsy with comorbid ataxia and cognitive impairment [84, 85]. Different lines of evidence support the notion that epilepsy is a condition characterized by network hyperexcitability. Epilepsy mutations are proposed to alter sodium channel behaviors by increasing the excitability of neurons expressing mutant channels. Consistently, studies have demonstrated the effects on sodium channel behavior [86, 87]. Amongst various types of GABAergic interneurons, NaV1.1 serves as the principal voltage-gated Na + channel. Decreased activity of NaV1.1 can reduce excitability and decrease the GABAergic tone. Mutations in NaV1.1 may be responsible for epilepsy. The potential of modulating the function of sodium channels has been increasingly supported by evidence as a potential therapeutic approach. Interneurons, which synthesize and release the inhibitory neurotransmitter GABA, are inhibitory in nature. They regulate the secure synchronized activity and the excitability of neuronal subpopulations. Categorization of interneurons into subclasses is determined by physiological properties, neurochemical markers, and connectivity patterns. For the parvalbumin-expressing subclass of inhibitory neurons (fast-spiking interneurons), the NaV1.1 channel significantlt contributes the sodium current, which is crucial for AP generation and sustained excitability. Therefore, specifically increasing the function of NaV1.1 channels can potentially enhance the function of fast-spiking GABAergic interneurons, leading to a consequential impact on the excitability in the central nervous system. Therefore, activation of NaV1.1 channels using pharmacological methods is considered as a viable treatment option for *SCN1A* haploinsufficiency and other diseases associated with defective function of fast-spiking GABAergic parvalbumin interneurons. It has been well established that the sodium channel is a crucial target of drugs. Small-molecule inhibitors targeting sodium channels have been deployed in clinical settings to treat various conditions linked with abnormal cellular excitability, such as epilepsy and pain, comprising the first generation of such inhibitors [88]. As the NaV1.1 channels are responsible for the modulation of electrical excitability through inhibitory interneurons, the use of non-selective sodium channel inhibitors is contraindicated to GEFS + syndromes or SMEI, as it might exacerbate the disease by further suppressing the NaV1.1 channels [89–91]. Clobazam, as the first-line drug therapy treating epilepsy associated with *SCN1A* mutations, which increases transmission of postsynaptic GABAergic signals with allosteric modulation of GABA_A_ receptors; and VPA, which increases GABA concentration in the synaptic gap through enhancement of GABA production and reduction of GABA degradation. Increasing the mRNA level of *SCN1A* using antisense nucleotides (ASO) has emerged as a promising approach for genetic disorders involving haploinsufficiency [92]. Alternative therapeutic options, such as ketogenic diets, may prove beneficial for cases of pharmacoresistant Dravet syndrome [93, 94]. Nav1.1 blockers as anti-seizure medications exert function by stabilizing neuronal membranes through inhibiting the initiation or propagation of abnormal synchronous electrical activity within neurons, thus attenuating the spread of seizure activity emerging from a particular focus or source [95]. Numerous studies have indicated that sodium channel blockers might be the optimum choice for individuals suffering from *SCN8A* encephalopathy [96–99].

Unlike *SCN1A*, where epilepsy stems almost exclusively from loss-of-function variants that impair channel function, which is caused by deficits in circuit disinhibition and inhibitory interneuron excitability [100–102], the *SCN2A* gene which encodes Nav1.2 is associated with seizures through both gain-of function and loss-of-function mutations. Nav1.2 is expressed mainly in excitatory pyramidal neurons, contrary to Nav1.1. Apart from seizure pathology, NaV1.2 loss-of-function mutations are also strongly associated with intellectual disability and autism spectrum disorder. More specifically, Nav1.2 is found in high density locating in the proximal region of axon initial segments (AIS),in which it is considered pivotal for the backpropagation of APs into the neuronal soma [103–105]. During embryonic development, at immature nodes of Ranvier, high levels of Nav1.2 exists. As the time of nodes maturation, Nav1.6 gradually supersedes Nav1.2 [106, 107]. Mutations in *SCN2A* are associated with inherited epilepsies including BFNIS. Specifically, in cases of BFNIS, the identification of missense mutations in *SCN2A* has been reported. [108]. Mutations in Nav1.2 also cause acquired form of seizures, such as febrile and afebrile seizures [109, 110]. Seizures caused by VGSC dysfunctions are intractable. In some cases, even various anti-seizure medications cannot controlled seizures caused by mutations of NaV1.2 [111]. Gain in channel function resulting from Nav1.2 mutations is believed to be a possible mechanism for epilepsy pathogenesis. Sodium channel inhibitors, such as carbamazepine, phenytoin, oxcarbazepine, lamotrigine and TPM, are generally expected to be effective in treating epilepsy patients who have a mutation in *SCN2A* (Nav1.2). Despite the success of carbamazepine and high dose of phenytoin in certain cases of epilepsy resulting from *SCN2A* mutations, a considerable number of patients remain pharmaceutically intractable, even when treated with other traditional anti-seizure medications. As a result, there is an urgent requirement for innovative, more targeted pharmaceutical agents for these patients. Peters, et al.investigated the effects of ranolazine, a pharmaceutical substance commercially utilized as an anti-anginal medication on NaV1.2 channels. This study found that ranolazine suppressed macroscopic currents and extended the recuperation time of both rapid and slow inactivation of NaV1.2 channels. Further consensus studies should be geared towards making ranolazine an approved and efficacious therapy for epilepsy but presently this is highly unlikely because it exacerbates seizure semiology [112]. It’s crucial to develop pharmacotherapeutic agents for epilepsy associated with pathogenic *SCN2A* mutation, which function primarily by amplifying resurgent and/or persistent currents, and may get involved in selective suppression of the aberrantly enhanced resurgent and/or persistent currents.

## Putative roles of Nav1.6 channel in both genetic and acquired epilepsies and its management.

The Nav1.6 channel encoded by the gene *SCN8A* is associated with over 300 cases of epileptic encephalopathy and ~ 200 putative spots of mutation have been characterized. Despite being one of the most massively expressed voltage-gated sodium channels in the CNS, Nav1.6 is the least studied of the Nav family. This gene has maximum expression in pyramidal cells of the hippocampal and the Purkinje cells and granule cells of the cerebellum [113]. This channel is particularly abundant in the distal part of AIS and in the nodes of Ranvier of myelinated axons, although it is also prevalent throughout the peripheral nervous systems and CNS, in both inhibitory and excitatory neurons [114]. Normal brain function is heavily reliant on the exquisite initiation and spread of APs, and this activity is crucially dependent on Nav1.6. Epilepsy-causing mutations in Nav1.6 occur through the entire structure of the channel and only 10% of these have been characterized at the molecular level. The majority of these mutations are gain-of-function mutations. Upregulation of Nav1.6 in the AIS is shown to result in an upsurge in spontaneous and repetitive firing of cortical neurons, a plausible explanation for why *SCN8A* mutations in patients with epilepsy are mainly gain-of-function and impact the AP threshold [115]. Although the function of Nav1.6 in inhibitory interneurons is still illusive, mounting evidence has indicated Nav1.6 plays a part in establishing synaptic inhibition within the thalamic network, corroborating the loss-of-function outcomes brought on missense mutations in the mature protein [116, 117], which result in various network effects in different circuits of the nervous system. There are three classical forms of hereditary epilepsies associated with mutations of *SCN8A*, early infantile epileptic encephalopathy type 13 (EIEE 13), benign familial infantile seizures-5 and paroxysmal dyskinesia with SUDEP, which is the primary cause of epilepsy-related death from mutations of *SCN8A*. One possible explanation for the relationship between *SCN8A*-related epilepsy and SUDEP is the broad expression of NaV1.6 in ventricular myocytes and cardiac tissues [118, 119]. Therefore, an accumulation of respiratory, neurological and cardiac factors lead to a “perfect storm” and thus result in death. Single and null mutations may have negative effect on the heart function, causing cardiorespiratory depression and, consequently, death [120]. EIEE 13 is a phenotypically complex early-onset epilepsy, with seizure onset before 18 months of age [121–123]. Examples of infantile epileptic encephalopathies are Lennox-Gastaut syndrome (LGS), Landau-Kleffner syndrome, myoclonic-astatic epilepsy, West syndrome, Ohtahara syndrome, and Dravet syndrome. Temporal lobe epilepsy (TLE) is one of the most common forms of adult-acquired epilepsy caused by gain-of-function mutations of *SCN8A*. Gain-of-function mutations of *SCN8A* are responsible for causing one of the most frequent types of acquired epilepsy (temporal lobe epilepsy, TLE) in adults. TLE can have myriad of etiologies. Seizures arising spontaneously from the temporal lobe are the hallmark feature of TLE. TLE is a complex and heterogeneous group of disorders with seizure initiation and invasion in the temporal lobe; however, variabilities exist amongst patients regarding their age of onset, etiologies, and response to various treatment approaches [124].

**Fig. 1:**
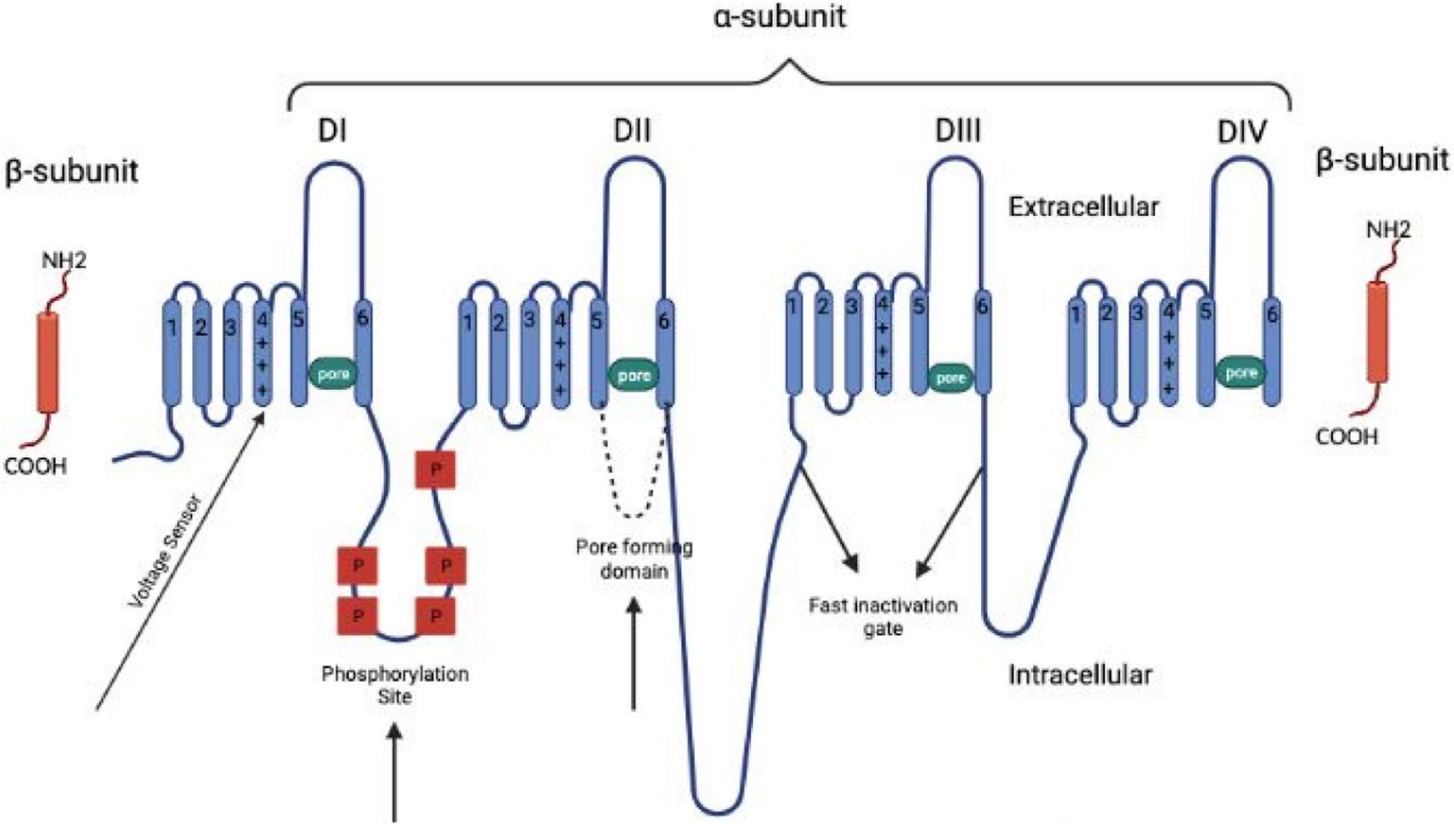
Primary structures of the subunits of voltage-gated sodium channel

It is well established that VGSCs make the substantial contribution in modulating neuronal proexcitatory and physiology changes to these channels facilitate neuronal hyperexcitability in TLE. In TLE patients, significant changes in VGSC mRNAs are observed in the hippocampus [125] and noteworthy recording resulting from human TLE subiculum neurons reveal a marked upsurge of persistent sodium currents [126]. Animal models of TLE have recapitulated corresponding proexcitatory modifications to sodium channels. 4,9- anhydro-tetrodotoxin (4,9-ah-TTX), a toxin with a more significant binding affinity for Nav1.6 compared to other VGSC isoforms, can reveal the critical function of Nav1.6 in promoting neuronal hyperexcitability [127]. Previous studies have shown that the inhibition of Nav1.6 with 4,9-ah-TTX can effectively dampen neuronal hyperexcitability and reduce upregulated persistent and resurgent sodium currents in TLE [128]. Due to the role in driving hyperexcitability of neurons, Nav1.6 has been an attractive target for preventing or decreasing the occurrence of seizures in TLE animals. For *SCN8A* encephalopathy, no guidelines for treatment is available. Current treatments are aimed to control seizures through poly-drug therapies, while uncontrolled seizures increase the risk of SUDEP and permanent injury in patients [129, 130]. Pharmacoresistance is typical among a number of *SCN8A* patients but some show prolonged seizure-free periods [131]. Many studies show that sodium channel inhibitors might be the most effective treatment approach for individuals with *SCN8A* encephalopathy [132]. A common pathogenic mechanism across *SCN8A* mutations is the disruption of sodium channel inactivation, and drugs that have greater affinity towards the inactivated state of these channels may offer advantage in treating patients. Phenytoin is one such drug that is believed to have higher affinity for the inactivated state [133]. Phenytoin can effectively attenuate proexcitatory alterations in the physiology of mutant channel [134] and offer improved seizure freedom in patients with *SCN8A* encephalopathy [135]. Although the treatment options with proven efficacy do exist for individuals with SCN8A encephalopathy, reports from several patients suggest that levetiracetam is ineffective at controlling seizures and may even worsen the symptoms [136]. TLE patients frequently exhibit pharmacoresistance and up to 30% of cases fail to attain seizure freedom only with ASMs [137, 138]. Recent studies have suggested that carbamazepine and valproate, inhibitors of sodium channel, are the most promising ASM options for TLE patients [139]. The efficacy of ASMs in treating individuals with TLE is limited by the modified pharmacology caused by epileptogenesis. In animal models of temporal lobe epilepsy, kindled animals show acute reduction of the hypoexcitatory effects of carbamazepine compared to controls. Additionally, the EC50 is significantly increased in cells from kindled animals compared to controls [140]. In brain tissues from carbamazepine-resistant TLE patients, the blocking effect of carbamazepine significantly diminished [141]. Further, the reduced effects of carbamazepine on the recovery from inactivation have been observed in animal models with TLE [142]. The reduced efficacy is not just seen with carbamazepine, but also with phenytoin and lamotrigine [143]. However, the effects of valproate exhibit little change in cells derived from epileptic patients and kindling animal models. [144]. More researches are needed to investigate if Nav1.6 is a promising target for future ASMs (Fig. 1).

## VGSC-inhibitors in the treatment of Dravet syndrome and other pharmacoresistant epilepsies

Although there is currently no cure for epilepsy, early treatment can lead to a substantial remission and make a big difference. Pharmacoresistance is a broad term that encompasses refractory, intractable or recalcitrant type of epilepsy such as Dravet syndrome, Ohtahara syndrome, Rasmussen encephalitis, LGS, and infantile spams. Pharmacoresistant epilepsy, as defined by The International League Against Epilepsy, refer to the failure of a patient to respond to at least two ASMs which are suitably chosen and adminisitered for an adequate period of time, either as a monotherapy or as a polytherapy [145–147]. The etiology of pharmacoresistant epilepsy can be attributed to various factors, comprising genetic and environmental factors, along with drug- and disease-related factors. Although about 30 ASMs have been approved for about three decades for the treatment of epilepsy, unfortunately some patients do not exhibit a positive response to medical interventions. Some VGSC blockers like lamotrigine and carbamazepine are the most effective and commonly used ASMs, they are surprisingly unable to cause remission in intractable epileptic encephalopathy like Dravet syndrome. In fact, they are contraindicated for Dravet syndrome because they exacerbate seizures [148, 149].

A pharmacoresistant form of epilepsy that emerges in infants, Dravet syndrome, leads to comorbidities of cognitive incapacity, psychomotor retardation, ataxia and premature mortality. Dravet syndrome represents the prototypical pharmacoresistant epilepsy. Seizures remain inadequately managed in a majority of patients, even with the use of multiple ASMs or polypharmacy. Aras et al. found that 45% of Dravet syndrome patients receiving inadequate treatment modalities persist in encountering over 4 tonic–clonic seizures each month [150]. Although Dravet syndrome is caused by polygenic mutations as mutations of other genes encoding calcium, potassium, and hyperpolarization-activated cyclic nucleotide-gated channels are also implicated in its pathogenesis, this disease is mainly caused by reduced sodium currents and impaired excitability of GABAergic interneurons (primarily defects in AP firing in fast-spike parvalbumin and somatostatin interneurons) in the hippocampus as well as to a lesser degree impairment of other classes of GABAergic interneurons. Agents that inhibit sodium channels are the drugs of choice for epileptic seizures, including lamotrigine, VPA, phenytoin, carbamazepine, and clobazam. Unfortunately, in fact, some of these standard sodium channel blockers worsen seizures in both mice and children with Dravet syndrome [151, 152]. Although some of them (such as VPA, TPM, rufinamide, cenobamate and eslicarbazepine) are effective for Dravet syndrome remission either as a monotherapy or in a polytherapy [153–155], most of the standard VGSC therapies are contraindicated in Dravet syndrome [156]. LGS is another important type of drug-resistant epilepsy. It is a rare and severe epileptic encephalopathy of childhood onset with heterogeneous etiology, in which 65–75% of patients have known causes (genetic, structural, or metabolic) while others have unknown causes [157, 158]. It is considered as one of the most severe and devastating type of epileptic syndromes in infancy and early childhood [159–161]. Even though VPA is not specifically licensed for application in LGS, owing to its broad spectrum and low potential for exacerbating seizures, it is widely recommended as an ideal first-line medicine [162, 163]. TPM is another broad-spectrum VGSC inhibitor that is used in the treatment of LGS. During long-term therapeutic studies, TPM demonstrated well tolerance and effectiveness in managing the drug-resistant drop attacks (sudden falls) and seizures linked with LGS [164, 165]. TPM is able to tackle seizure semiologies like tonic seizures, characterized by greatly increased muscle tone and abrupt stiffening movement in the limbs and body, which is commonly observed in LGS, as well as atonic seizures that manifest as sudden loss in muscle strength and tone [165–170]. Most of the ASMs available are used in polytherapies in the management of LGS, and emerging drugs are being re-directed to develop LGS-specific treatments. As per recent research, rufinamide is among the latest adjunctive drugs [171–174]. Both open-label studies and randomized controlled trials have suggested that rufinamide could be highly efficacious in mitigating a range of seizures, especially tonic-atonic seizures and those leading to falls observed in patients with LGS. Rufinamide appears to have an advantageous tolerability and safety profile, together with largely mild side effects as well as a good interaction profile with other ASMs. Other VGSC inhibitors in the treatment of LGS include ZNS, which is indicated as adjunctive safe and effective treatment in pediatric LGS patients [175–178]. Lamotrigine (LCM) was specifically approved for the management of LGS by US and EU after a double-blind, placebo-controlled, randomized controlled trial, which certified that LCM is efficacious in the treatment of LGS [179, 180]. According to recent research, the application of LCM exhibits the potential to decrease the huge number of spike-and-wave events that are commonly observed in LGS [181, 182], and also demonstrates efficacy as an adjunctive therapy for treating refractory epilepsy [183]. Although phenytoin can exacerbate atypical absences and myoclonic-seizures in LGS [184], it still plays a pivotal role in the management of LGS as it decreases tonic–clonic seizures and reduces tonic seizures.

Another pharmacoresistant type of epilepsy that can benefit from treatment with VGSC inhibitors is the early infantile epileptic encephalopathy (EIEE). EIEE is a childhood age-dependent disease of the brain, with pathological hallmarks including loss of neurologic function over time, abnormal electroencephalographic findings, and seizures. Although EIEE seizures are devastating, debilitating, intractable and pharmacoresistant to ASMs, some patients respond positively to high-dose VPA [185]. VPA also showed good efficacy in a EIEE patient caused by *PACS2* gene mutation [186]. Unfortunately, however, VPA did not show beneficial effect in early diagnosis and treatment of an infant with epileptic encephalopathy caused by cytoplasmic FMRP interacting protein 2 mutation [187]. VGSC inhibitors are typically considered the first-line treatment for confirmed or suspected epileptic encephalopathies related to *SCN2A*. In severe cases with compatible electro-clinical features, carbamazepine used following a high-dose intravenous phenytoin (in the case of a positive response to phenytoin) may be a more suitable treatment algorithm for long-term maintenance treatment [188]. Carbamazepine as a VGSC inhibitor is also effective for RHOBTB2-related paroxysmal dyskinesia resulting from early infantile *SCN1A* epileptic encephalopathy [188] (Table 1).

**Table 1:** Therapeutic relevance of VGSC inhibitors in the treatment of epilepsies, DS and other pharmacoresistant epilepsies, and limitations of specific inhibitors

VGSC inhibitor	Form of epilepsy	Clinical efficacy	Limitation of inhibitors
**Phenytoin**	Status epilepticus	More efficacious than levetiracetam in treating SE	Exacerbate Dravet syndrome and other *SCNA1* related epilepsies
	EIEE	Most effective in treatment of EIEE	Aggravate Juvenile myoclonic epilepsy
**Rufinamide**	LGS	Adjunctive for the treatment of LGS	Numerous adverse side effects: Hyperkinesia, hypotension, locomomotor dysfunction, megaloblastic anemia, SJS, hypotension, hyperkinesia, cardiovascular collapse, peripheral neuropathy
	EIMFS	Effective in treating migrating focal seizures	
	*SCN8A* encephalopathy	Effective against *SCN8A* encephalopathy Mitigate *SCN1B* mutation-related epilepsy	
		(Epileptic encephalopathy, early infantile, EIEE52)	
		Moderate efficacy in treatment of benign familial infantile, EIEE5	
	GTCS	Used to treat general tonic-colonic seizures	
	CPS	Remission of complex partial seizures	
	Myoclonic seizures	PHT should not be used to treat myoclonic seizures	
**Carbamazepine**	*SCN2A* epilepsy	Effective in some *SCN2A* epileptic	May aggravate Juvenile myoclonic epilepsy and DS
		Patients at high doses	
	TLE	Most effective against TLE	Exacerbate generalized atypical absence seizure
	Bilateral tonic clonic seizure	Ameliorate bilateral tonic clonic seizure	
	Simple partial seizure	Remission of simple partial seizure	Some side effects may include:
	Tonic–clonic seizure	Alleviate Tonic–clonic seizures	Blurry or double vision
	DS	Contraindicated in DS	Nausea, headache, dizziness
**Valproic acid**	Juvenile myoclonic epilepsy	Control of seizures in 70% to 85% of patients treated with valproic acid monotherapy or polytherapy	VPA therapy induces hypoadiponectinemia which correlates negatively with insulin resistance
	DS	First-line agent to cause remission in DS but not effective to cause total seizure freedom	
	Lennox-Gastaut syndrome	One of the first-line therapies for the treatment of Lennox-Gastaut Syndrome	Induced weight, seems to be appetite-related than metabolic
	Temporal lobe epilepsy	Effective in the treatment of TLE when combined with CBZ and PTH	potent teratogen
	Myoclonic seizures	Often exquisitely responsive to treatment with valproic	Rarely used in the elder ly because of its hepatotoxic effects
	Refractory seizures	Causes seizure freedom in patients with refractory seizures	
	Tonic–clonic seizures	Effective for primary generally tonic–clonic seizures compared to lamotrigine	Its use is accompanied with numerous side effects:
	Focal aware seizure	Relative efficacious in the treatment of FAS	VPA encephalopathy, coagulopathies, diarrhea, vomiting, bone marrow anomaly ataxia, tremor
	Bilateral tonic clonic seizure	Equal efficacy with ethosuximide but more efficacious than lamotrigine in the management of BTCS	Valproic acid-induced hyperammonemia in children
	Complex partial seizures	Effective in the control of CPS	
**Lamotrigine**	Subtle seizures/Focal seizures	Very favorable drug for treating epilepsy in people with intellectual disability	Highly tolerable with few adverse side effects: diplopia, headache, ataxia
	DS	LCM is contraindicated in DS	
	Refractory Seizures/GTS/ Absence	Lamotrigine has certain advantages over Carbamazepine because it has less side effects and high tolerability	
	LGS	Cause remission in patients with LGS	
	Myoclonic Seizures	LCM aggravates myoclonic seizures	
**TPM**	GTCS/Absence/Focal seizures	Has efficacy as monotherapy or adjunctive therapy in adult and paediatric patients with GTCS, absence and focal seizures	Mild side effect of cognitive impairment
	Myoclonic seizures	TPM appears effective in neonatal seizure Like myoclonic seizure	
	LGS	Adjunctive therapy in patients with LGS	Undefined sleep disturbances
	Dravet syndrome	TPM is useful as an adjunctive therapy in patients with DS and carry additional benefits in other refractory cases of epilepsies	
**Oxcarbazepine**	Partial-onset seizures	Effective in alone or in combination with other ASMs in the treatment of partial-onset seizures	Fewer side effects compared to its other family member of CBZ and ESL
	GTCS	Monotherapy is as effective as phenytoin and vaiproic acid at reducing generalised tonic–clonic and partial seizure frequency	Side effects include: hyponatremia, thyroid abnormalities double vision, mental-depression, crying, clumsiness
		more favorable pharmacokinetic and metabolic profile when used in the treatment of GTCS	
	Myoclonic seizures	Oxcarbazepine worsen myoclonic seizures	Should be avoided in patients with myoclonic
	Dravet syndrome	should be avoided, as they typically exacerbate DS type of seizure	
**Eslicarbazepine**	TLE	More effective in reducing TLE seizure frequency than a placebo	Similar side effects with other family members of CBZ and ZNS such as:
	FIAS	Suppress seizure activity but may also inhibit the generation of a hyperexcitable network	Diplopia
	SGS	Effective, safe and well tolerated third-generation ASM for the treatment of focal epilepsies	
	DS	Beneficial in some DS patients	
**Zonisamide**	LGS	Effective due to its broad spectrum of anticonvulsant activity	
	FIAS	Long-term use of adjunctive zonisamide therapy may be beneficial with FIAS	
	Dravet syndrome	Fails to decrease seizures in all *SCN1A*-positive cases	
**Lacosamide**	Focal aware seizure/GTCS	Favorable tolerability and pharmacokinetic profiles when used in the treatment of partial-onset seizures and generalized tonic–clonic seizures	LCM therapy has a higher level of suicidal thought and actions
	MTLE	Early LCM treatment has effective anti-ictogenic properties in the pilocarpine model of MTLE	Increase chances of development of atrial fibrillation
	DS	Not effective in tackling DS	
	SPS/ idiopathic generalized epilepsy	Adjunctive treatment for uncontrolled primary generalized tonic–clonic seizures in those with idiopathic generalized epilepsy	
	DRE	Effective in the treatment of focal DRE	Rarely, a drop in White blood cell is Seen in patients
**Fosphenytoin**	GTCS	Alternative to intravenous phenytoin for acute. treatment of seizures	
		Advantages include more convenient and rapid intravenous administration, availability for intramuscular injection, and low potential for adverse local reactions at injection sites	Drawbacks include the occurrence of transient paraesthesias and pruritus at infusion rates and cost
	DS	Just like its prodrug phenytoin, Fosphenytoin should be avoided as medication for DS but may be useful in the treatment of SE	
**Cenobamate**	DS	May lead to a clinically meaningful reduction of seizure frequency in a few adult patients with DS	It is unclear, if all patients with DS benefit, requiring further investigations and functional experiments
	Focal-onset seizures	Effective in the treatment of focal-onset seizures	Complex drug–drug interactions which decrease plasma concentrations of LCM and CBZ and increase levels of PHT
	LGS	CNB may represent a promising therapeutic option in patients with drug-resistant epilepsy associated with LGS	

*EIEE* Early infantile epileptic encephalopathy, *SE* Status epilepticus, *EIMFS* Epilepsy of infancy with migrating focal seizures, *GTCS* Generalized tonic–clonic seizure, *CPS* Complex partial seizures, *TLE* Temporal lobe epilepsy, *PHT* Phenytoin, *VPA* Valproic acid, *CBZ* Carbamazepine, *PTH* Phenytoin, *BTCS* Bilateral Tonic Clonic Seizure, *LCM* Lamotrigine, *GTCS* Generalized tonic–clonic seizures, *FIAS* Focal impaired awareness seizures, *SGS* Secondary generalized seizure, *LCM* Lacosamide, *MTLE* Mesial temporal lobe epilepsy, *DRE* Drug resistant epilepsy, *CNB* Cenobamate

## Monotherapy vs. rational polytherapy with VGSC inhibitors in the management of pharmacoresistant and other forms of epilepsy

At present, treatment selections for epilepsy primarily addresses symptoms. After the first two drug regimens, most patients can achieve seizure freedom. Otherwise, they are defined as pharmacoresistant. Due to the various benefits including minimal side-effects, lack of drug-drug interactions, better adherence and lower cost, monotherapy is considered as the preferable treatment approach in epilepsy [189]. Another major impetus to achieve or maintain monotherapy is the fact that it decreases addictive neurotoxic and cognitive side effects. Carbamazepine and VPA are two well-known effective monotherapeutic ASMs and have been in use for the management of seizures for decades with remarkable success. One of the initial ASMs to be promoted for application as monotherapy was VPA. In a previously open monotherapy investigation, VPA was found to be effective in regulating all primary generalized seizure types in 83% of 118 patients evaluated in both adults and children, some of whom had failed to respond to the former treatment [190]. Carbamazepine has also robustly demonstrated effectiveness in monotherapy for seizures. Another dibenzazepine family member, eslicarbazepine acetate, has been sanctioned by the European Medicines Agency (EMA) and United States Food and Drug Administration (FDA) for monotherapy in adults with newly diagnosed epilepsy [191–194]. Apart from the aforementioned VGSC inhibitors, several other inhibitors have also demonstrated their potency and efficacy as monotherapeutic agents against epilepsy. Unfortunately, drug-resistant epilepsy occurs in at least 30% of people with epilepsy, who remain refractory to traditional pharmacological therapies, necessitating multiple drugs to be used simultaneously [195, 196]. In addition, despite the introduction of new ASMs over the past two decades with advances in the field, management of pharmacoresistant epilepsies is still complicated and leaves a lot of unsolved questions. Also, a study has demonstrated that 30–40% of patients treated with an traditional ASM including carbamazepine and VPA as monotherapy experience adverse effects that contribute to therapeutic failure [197]. Over the past 20 years, monotherapy has been considered as the gold standard in epilepsy treatment, partly due to the heightened toxicity associated with polytherapy. Nevertheless, some people with pharmacoresistant epilepsy, such as LGS and Dravet syndrome, have not shown expected response to monotherapy. Such patients may require polytherapy and should be carefully evaluated taking into account the risk/benefit ratio in terms of tolerability, potency/efficacy and patient compliance. Rationally designed polytherapies can achieve better seizure control, maximize the efficacy, minimize drug interactions, drug load, and side effects, and control multiple seizure types that react to various therapeutic drugs [198, 199].

The aims of using polytherapy in pharmacoresistant and other types of epilepsy are to maximize efficacy and minimize side effects [200]. Another aim of polytherapy and its practice as the first-line of treatment for refractory epilepsy is to achieve robust synergistic impact or lower drug toxicity with less doses of two medications instead of higher doses of a single drug. [201]. The usefulness of combination therapy should be an anti-seizure supra-additive effect (synergy effect) and possibly neurotoxic antagonism or neurotoxic infra-additive effect [202].

Polytherapy is highly desirable in the treatment of pharmacoresistant and other forms of epilepsies. In many non-randomized open studies, the efficacy of CBZ and VPA in combination has been established in patients who exhibited poor response to monotherapy [203, 204]. Robust data have shown that the combination of VPA and lamotrigine exerts the best synergism in human studies. Multiple studies have reported on the synergistic relationship between these anti-seizure medications, highlighting the substantial response rate generated through the incorporation of lamotrigine as an add-on therapy to VPA, in contrast to addition of lamotrigine to phenytoin or carbamazepine [205]. Polytherapy of LTG-TPM and VPA is also useful in adults [206–208]. Combinational therapies of trio-ASMs including zonisamide, gabapentin, and eslicarbazepine acetate have also shown to be effective as additional drugs in treating drug-resistant epilepsy [209–211]. VPA, a VGSC inhibitor, is still considered as the primary ASM treating recently diagnosed drug-resistant epilepsy like DS and LGS. If VPA fails to cause seizure freedom, then another VGSC inhibitor lamotrigine can be considered. Duotherapies combining other VGSC inhibitors like rufinamide and TPM have shown effectiveness against LGS and drop attacks [168, 212–215]. The combinational therapy of TPM and CLB has also shown effectiveness. FFA, perampanel, LEV and zonisamide may have an efficacy in LGS [216]. Polytherapy holds the future ace in the management of epilepsy, especially drug-resistant epilepsy, if the therapeutic factors and variables are strictly adhered to during these combinations. A recent review proposes that novel ASMs are preferred candidates for combinational therapy as they possess fewer pharmacokinetic interactions, predominantly weak enzyme inhibitors or inducers, and exhibit superior tolerability profiles [217]. Based on this, ASMs with fewest pharmacological interactions (TPM and zonisamide) are the best to be used in polytherapy for optimal results and few adverse events. In fact, some researchers propose that the occurrence of adverse events in polytherapy is not solely linked to the higher number of drugs but rather to the type and the dosage of the ASMs as well as individual vulnerability [218, 219]. In addition, physicians should consider epilepsy syndromes and seizure types before selecting the best combination of ASMs. It is also crucial to take into account various other factors, such as pharmacokinetic and pharmacodynamic aspects of anti-seizure medications, along with patient-related elements including concomitant medications, pharmacogenomics, age, comorbidities, and compliance. It is pertinent to state that the well-defined pharmacoresistant epilepsies such as DS, LGS, EIEE and Rasmussen encephalitis need combinational therapies (such as polytherapy of TPM and VPA) especially where monotherapy is unable to lead to remission.

## Conclusions

ASMs that inhibit VGSCs represent a fundamental aspect of treating epilepsy. Manipulations of VGSCs are the primary and most important mechanisms through which virtually all ASMs exert their antiepileptic potentials. Experimental data and evidence gathered over the past two to three decades suggest that VGSC inhibitors acting by blockade of sodium channels are the most effective ASMs in use today. In addition, some other ASMs whose main mechanisms of action are not directly related to VGSCs, have been shown to interact with voltage-gated sodium currents, called multimodality therapy that could be efficacious in the treatment of pharmacoresistant and other types of epilepsies. Presently available ASMs that function through inhibition of VGSCs are often effective in controling seizures in many patients. Unsurprisingly, however, seizure freedom is not totally achieved as seizures persist in a large number of epileptic patients due to drug resistance. Pharmacoresistant epilepsies are a type of highly refractory epilepsies in which a substantial proportion (about 30%) of epilepsy individuals exhibit treatment resistance to any of the three first-line ASMs, despite being administered in an optimal and monitored regimen. Despite efforts by epileptologists and other related researchers to unravel the molecular and cellular mechanisms underpinning pharmacoresistant epilepsies, a complete understanding has so far remained elusive. For the majority of the patients with epilepsy, polytherapy is still the reality. However, investigations conducted in animal models have not revealed evidently about the mechanisms underlying the effectiveness of polytherapy in humans, epileptologists may engage in pharmacogenomics that might provide other instructions as to which combinations or polytherapy could be efficacious potentially via the development of personalized therapeutic plans. Some VGSC inhibitors are not only ineffective in treating some forms of pharmacoresistant epilepsies as seen in DS and LGS, but are also contraindicated as they worsen seizures. The ineffectiveness of VGSC inhibitors in pharmacoresistance epilepsy like DS is not too surprising considering the fact that the pathogenesis of DS is not purely a consequence of epilepsy but is precipitated as a result of genetic mutations encoding mainly VGSCs and other channels like potassium and calcium channels modulated by genetic and non-genetic factors. In an effort to treat and mitigate the burden of these pharmacoresistant epilepsies, some combinatorial have all been approved by relevant regulatory bodies (FDA & EMA) for the treatment of pharmacoresistant epilepsies. Although VGSC inhibitors remain the standard therapy and mainstay in epilepsy treatments over the years, present VGSC inhibitors have discrimination among different VGSC isoforms, because of polygenic and heterogenous nature and thus selective blockers development might enhance their clinical utility. Therefore, there is a need to develop novel drug cocktails with higher selectivity for specific VGSC subtypes, which may be effective in treating several types of epileptic seizures. Despite recent breakthroughs, with approval of the polytherapy treatments for pharmacoresistant epilepsies, patients still encounter significant chanllenges due to the multifactorial nature and limited understanding of pathogenesis and mechanisms of these pharmacoresistant epilepsies. Therefore, more studies are needed to advance our understanding of the pathogenesis of pharmacoresistant epilepsies, which could provide further insight into their precise treatment. Also, research efforts should gear towards discovery, designing and development of optimal combination of VGSC blockers to achieve the maximum therapeutic effectiveness with minimum side effects. Alternatively, non-pharmacological methods such as ketogenic diet therapy and electrical stimulation are also showing emerging potentials.

## Data Availability

Not applicable.

## Abbreviations

ADNFLE: Autosomal dominant nocturnal frontal lobe epilepsy
ASM: Anti-seizure medication
AMPA: α-Amino-3-hydroxy-5-methyl-4-isoxazolepropionic acid
BFNIS: Benign familial neonatal infantile seizures
BFNS: Benign familial neonatal seizures
DS: Dravet syndrome
GABA: γ-Aminobutyric acid
GEFS +: Genetic epilepsy with febrile seizures plus
IFM: Isoleucine-phenylalanine-methionine
IGE: Idiopathic genetic epilepsies
INaP: Persistent sodium currents
LGIC: Ligand gated ion channel
LGS: Lennox Gastuat syndrome
nAChRs: Nicotinic acetylcholine receptors
PKA: CAMP-dependent protein kinase
PKC: Protein Kinase C
PTM: Post-translational modification
SMEI: Serve myoclonic epilepsy in infancy
TRPV1: Transient receptor potential vanilloid-1
VGIC: Voltage- gated ion channel
VGSC: Voltage- gated sodium channel
VSD: Voltage-sensing domain

## References

[1] Lozovaya N, Gataullina S, Tsintsadze T, Tsintsadze V, Pallesi-Pocachard E, Minlebaev M, et al. Selective suppression of excessive GluN2C expression rescues early epilepsy in a tuberous sclerosis murine model. Nat Commun. 2014;5:4563. 25081057 10.1038/ncomms5563 PMC4143949

[2] Goldenberg MM. Overview of drugs used for epilepsy and seizures: etiology, diagnosis, and treatment. P T. 2010;35(7):392–415. 20689626 PMC2912003

[3] Jacoby A, Snape D, Baker GA. Epilepsy and social identity: the stigma of a chronic neurological disorder. Lancet Neurol. 2005;4(3):171–8. 15721827 10.1016/S1474-4422(05)01014-8

[4] Rincon N, Barr D, Velez-Ruiz N. Neuromodulation in drug resistant epilepsy. Aging Dis. 2021;12(4):1070. 34221550 10.14336/AD.2021.0211 PMC8219496

[5] Yemadje LP, Houinato D, Quet F, Druet-Cabanac M, Preux PM. Understanding the differences in prevalence of epilepsy in tropical regions. Epilepsia. 2011;52(8):1376–81. 21627649 10.1111/j.1528-1167.2011.03099.x

[6] Stafstrom CE, Carmant L. Seizures and epilepsy: an overview for neuroscientists. Cold Spring Harb Perspect Med. 2015;5(6):a022426. 26033084 10.1101/cshperspect.a022426 PMC4448698

[7] Guerrini R, Marini C, Mantegazza M. Genetic epilepsy syndromes without structural brain abnormalities: clinical features and experimental models. Neurotherapeutics. 2014;11:269–85. 24664660 10.1007/s13311-014-0267-0 PMC3996114

[8] Lerche H, Shah M, Beck H, Noebels J, Johnston D, Vincent A. Ion channels in genetic and acquired forms of epilepsy. J Physiol. 2013;591(4):753–64. 23090947 10.1113/jphysiol.2012.240606 PMC3591694

[9] Mantegazza M, Catterall WA. Voltage-Gated Na+ Channels: Structure, Function, and Pathophysiology. 4th ed. Jasper’s Basic Mechanisms of the Epilepsies. 2012. 22787615

[10] Catterall WA, Swanson TM. Structural basis for pharmacology of voltage-gated sodium and calcium channels. Mol Pharmacol. 2015;88(1):141–50. 25848093 10.1124/mol.114.097659 PMC4468632

[11] Catterall WA. From ionic currents to molecular mechanisms: the structure and function of voltage-gated sodium channels. Neuron. 2000;26(1):13–25. 10798388 10.1016/s0896-6273(00)81133-2

[12] Beneski DA, Catterall WA. Covalent labeling of protein components of the sodium channel with a photoactivable derivative of scorpion toxin. Proc Natl Acad Sci U S A. 1980;77(1):639–43. 6928649 10.1073/pnas.77.1.639 PMC348330

[13] Yu FH, Catterall WA. Overview of the voltage-gated sodium channel family. Genome Biol. 2003;4(3):207. 12620097 10.1186/gb-2003-4-3-207 PMC153452

[14] Shi X, Yasumoto S, Nakagawa E, Fukasawa T, Uchiya S, Hirose S. Missense mutation of the sodium channel gene SCN2A causes Dravet syndrome. Brain and Dev. 2009;31(10):758–62. 10.1016/j.braindev.2009.08.009 19783390

[15] O’Malley HA, Isom LL. Sodium channel β subunits: emerging targets in channelopathies. Annu Rev Physiol. 2015;10(77):481–504. 10.1146/annurev-physiol-021014-071846 PMC4817109 25668026

[16] Catterall WA. Voltage-gated sodium channels at 60: structure, function and pathophysiology. J Physiol. 2012;590(11):2577–89. 22473783 10.1113/jphysiol.2011.224204 PMC3424717

[17] de Lera RM, Kraus RL. Voltage-gated sodium channels: structure, function, pharmacology, and clinical indications. J Med Chem. 2015;58(18):7093–118. 25927480 10.1021/jm501981g

[18] Noda M, Hiyama TY. The Na(x) channel: what it is and what it does. Neuroscientist. 2015;21(4):399–412. 24962095 10.1177/1073858414541009

[19] Clairfeuille T, Cloake A, Infield DT, Llongueras JP, Arthur CP, Li ZR, et al. Structural basis of α-scorpion toxin action on Nav channels. Science. 2019;363(6433):eaav8573. 30733386 10.1126/science.aav8573

[20] Catterall WA, Goldin AL, Waxman SG. International Union of Pharmacology. XLVII. Nomenclature and structure-function relationships of voltage-gated sodium channels. Pharmacol Rev. 2005;57(4):397–409. 16382098 10.1124/pr.57.4.4

[21] Chung S, Skinner J, Rees M. Molecular genetics of arrhythmias. Principles and Practice of Clinical Cardiovascular Genetics. Oxford: Oxford University Press; 2010. p. 239–49.

[22] Lupoglazoff JM, Cheav T, Baroudi G, Berthet M, Denjoy I, Cauchemez B, et al. Homozygous SCN5A mutation in long-QT syndrome with functional two-to-one atrioventricular block. Circ Res. 2001;89(2):E16-21. 11463728 10.1161/hh1401.095087

[23] Baroudi G, Chahine M. Biophysical phenotypes of SCN5A mutations causing long QT and Brugada syndromes. FEBS Lett. 2000;487(2):224–8. 11150514 10.1016/s0014-5793(00)02360-7

[24] Baroudi G, Carbonneau E, Pouliot V, Chahine M. SCN5A mutation (T1620M) causing Brugada syndrome exhibits different phenotypes when expressed in Xenopus oocytes and mammalian cells. FEBS Lett. 2000;467(1):12–6. 10664447 10.1016/s0014-5793(00)01099-1

[25] Rivolta I, Abriel H, Tateyama M, Liu H, Memmi M, Vardas P, et al. Inherited Brugada and long QT-3 syndrome mutations of a single residue of the cardiac sodium channel confer distinct channel and clinical phenotypes. J Biol Chem. 2001;276(33):30623–30. 11410597 10.1074/jbc.M104471200

[26] Isom LL, De Jongh KS, Catterall WA. Auxiliary subunits of voltage-gated ion channels. Neuron. 1994;12(6):1183–94. 7516685 10.1016/0896-6273(94)90436-7

[27] Brackenbury WJ, Isom LL. Na+ Channel β Subunits: Overachievers of the Ion Channel Family. Front Pharmacol. 2011;2:53. 22007171 10.3389/fphar.2011.00053 PMC3181431

[28] Chahine M, O’Leary ME. Regulatory role of voltage-gated Na+ channel β subunits in sensory neurons. Front Pharmacol. 2011;2:70. 22125538 10.3389/fphar.2011.00070 PMC3221288

[29] Namadurai S, Balasuriya D, Rajappa R, Wiemhöfer M, Stott K, Klingauf J, et al. Crystal structure and molecular imaging of the Nav channel β3 subunit indicates a trimeric assembly. J Biol Chem. 2014;289(15):10797–811. 24567321 10.1074/jbc.M113.527994 PMC4036194

[30] Waxman SG. Axonal conduction and injury in multiple sclerosis: the role of sodium channels. Nat Rev Neurosci. 2006;7(12):932–41. 17115075 10.1038/nrn2023

[31] Freitag CM, May TW, Pfäfflin M, König S, Rating D. Incidence of epilepsies and epileptic syndromes in children and adolescents: a population-based prospective study in Germany. Epilepsia. 2001;42(8):979–85. 11554882 10.1046/j.1528-1157.2001.042008979.x

[32] Steinlein OK. Genetic mechanisms that underlie epilepsy. Nat Rev Neurosci. 2004;5(5):400–8. 15100722 10.1038/nrn1388

[33] Weber YG, Lerche H. Genetic mechanisms in idiopathic epilepsies. Dev Med Child Neurol. 2008;50(9):648–54. 18754913 10.1111/j.1469-8749.2008.03058.x

[34] Wallace RH, Wang DW, Singh R, Scheffer IE, George AL, Phillips HA, et al. Febrile seizures and generalized epilepsy associated with a mutation in the Na+-channel ß1 subunit gene SCN1B. Nat Genet. 1998;19(4):366–70. 9697698 10.1038/1252

[35] Buchhalter JR. Animal models of inherited epilepsy. Epilepsia. 1993;34:S31-41. 8500431 10.1111/j.1528-1167.1993.tb06257.x

[36] Stein RE, Kaplan JS, Li J, Catterall WA. Hippocampal deletion of NaV1. 1 channels in mice causes thermal seizures and cognitive deficit characteristic of Dravet Syndrome. Proc Natl Acad Sci U S A. 2019;116(33):16571–6. 31346088 10.1073/pnas.1906833116 PMC6697805

[37] Oyrer J, Maljevic S, Scheffer IE, Berkovic SF, Petrou S, Reid CA. Ion channels in genetic epilepsy: from genes and mechanisms to disease-targeted therapies. Pharmacol Rev. 2018;70(1):142–73. 29263209 10.1124/pr.117.014456 PMC5738717

[38] Steinlein OK, Mulley JC, Propping P, Wallace RH, Phillips HA, Sutherland GR, et al. A missense mutation in the neuronal nicotinic acetylcholine receptor α4 subunit is associated with autosomal dominant nocturnal frontal lobe epilepsy. Nat Genet. 1995;11(2):201–3. 7550350 10.1038/ng1095-201

[39] Alabi AA, Bahamonde MI, Jung HJ, Kim JI, Swartz KJ. Portability of paddle motif function and pharmacology in voltage sensors. Nature. 2007;450(7168):370–5. 18004375 10.1038/nature06266 PMC2709416

[40] Stafstrom CE. Persistent sodium current and its role in epilepsy. Epilepsy Curr. 2007;7(1):15–22. 17304346 10.1111/j.1535-7511.2007.00156.x PMC1797888

[41] Eijkelkamp N, Linley JE, Baker MD, Minett MS, Cregg R, Werdehausen R, et al. Neurological perspectives on voltage-gated sodium channels. Brain. 2012;135(9):2585–612. 22961543 10.1093/brain/aws225 PMC3437034

[42] Oliva M, Berkovic SF, Petrou S. Sodium channels and the neurobiology of epilepsy. Epilepsia. 2012;53(11):1849–59. 22905747 10.1111/j.1528-1167.2012.03631.x

[43] Marini C, Scheffer IE, Nabbout R, Suls A, De Jonghe P, Zara F, et al. The genetics of Dravet syndrome. Epilepsia. 2011;52:24–9. 21463275 10.1111/j.1528-1167.2011.02997.x

[44] Fujiwara T, Sugawara T, Mazaki-Miyazaki E, Takahashi Y, Fukushima K, Watanabe M, et al. Mutations of sodium channel α subunit type 1 (SCN1A) in intractable childhood epilepsies with frequent generalized tonic–clonic seizures. Brain. 2003;126(3):531–46. 12566275 10.1093/brain/awg053

[45] DeLorenzo RJ, Sun DA, Deshpande LS. Erratum to “Cellular mechanisms underlying acquired epilepsy: the calcium hypothesis of the induction and maintenance of epilepsy.” Pharmacol Ther. 2005;105(3):229–66 Pharmacol Ther. 2006;111(1):288-325. 16832874 10.1016/j.pharmthera.2004.10.015

[46] Wengert ER, Patel MK. The role of the persistent sodium current in epilepsy. Epilepsy Curr. 2021;21(1):40–7. 33236643 10.1177/1535759720973978 PMC7863310

[47] Stafstrom CE. Severe epilepsy syndromes of early childhood: the link between genetics and pathophysiology with a focus on SCN1A mutations. J Child Neurol. 2009;24(8_suppl):15S-23S. 19666879 10.1177/0883073809338152

[48] Maljevic S, Lerche H. Potassium channels: a review of broadening therapeutic possibilities for neurological diseases. J Neurol. 2013;260:2201–11. 23142946 10.1007/s00415-012-6727-8

[49] Rogawski MA, Löscher W. The neurobiology of antiepileptic drugs. Nat Rev Neurosci. 2004;5(7):553–64. 15208697 10.1038/nrn1430

[50] Sills GJ, Rogawski MA. Mechanisms of action of currently used antiseizure drugs. Neuropharmacology. 2020;168:107966. 32120063 10.1016/j.neuropharm.2020.107966

[51] Brodie MJ. Sodium channel blockers in the treatment of epilepsy. CNS Drugs. 2017;31(7):527–34. 28523600 10.1007/s40263-017-0441-0

[52] Kwan P, Arzimanoglou A, Berg AT, Brodie MJ, Allen Hauser W, Mathern G, et al. Definition of drug resistant epilepsy: consensus proposal by the ad hoc Task Force of the ILAE Commission on Therapeutic Strategies. Epilepsia. 2010;51(6):1069–77. 19889013 10.1111/j.1528-1167.2009.02397.x

[53] Khoury GA, Baliban RC, Floudas CA. Proteome-wide post-translational modification statistics: frequency analysis and curation of the swiss-prot database. Sci Rep. 2011;1:90. 22034591 10.1038/srep00090 PMC3201773

[54] Berendt FJ, Park KS, Trimmer JS. Multisite phosphorylation of voltage-gated sodium channel α subunits from rat brain. J Proteome Res. 2010;9(4):1976–84. 20131913 10.1021/pr901171q PMC2849892

[55] Baek JH, Cerda O, Trimmer JS. Mass spectrometry-based phosphoproteomics reveals multisite phosphorylation on mammalian brain voltage-gated sodium and potassium channels. Semin Cell Dev Biol. 2011;22(2):153–9. 20932926 10.1016/j.semcdb.2010.09.009 PMC3043121

[56] Li M, West JW, Numann R, Murphy BJ, Scheuer T, Catterall WA. Convergent regulation of sodium channels by protein kinase C and cAMP-dependent protein kinase. Science. 1993;261(5127):1439–42. 8396273 10.1126/science.8396273

[57] West JW, Numann R, Murphy BJ, Scheuer T, Catterall WA. A phosphorylation site in the Na+ channel required for modulation by protein kinase C. Science. 1991;254(5033):866–8. 1658937 10.1126/science.1658937

[58] Numann R, Catterall WA, Scheuer T. Functional modulation of brain sodium channels by protein kinase C phosphorylation. Science. 1991;254(5028):115–8. 1656525 10.1126/science.1656525

[59] Li M, West JW, Lai Y, Scheuer T, Catterall WA. Functional modulation of brain sodium channels by cAMP-dependent phosphorylation. Neuron. 1992;8(6):1151–9. 1319185 10.1016/0896-6273(92)90135-z

[60] Catterall WA, Perez-Reyes E, Snutch TP, Striessnig J. International Union of Pharmacology. XLVIII. Nomenclature and structure-function relationships of voltage-gated calcium channels. Pharmacol Rev. 2005;57(4):411–25. 16382099 10.1124/pr.57.4.5

[61] Cantrell AR, Catterall WA. Neuromodulation of Na+ channels: An unexpected form of cellular platicity. Nat Rev Neurosci. 2001;2(6):397–407. 11389473 10.1038/35077553

[62] James TF, Nenov MN, Wildburger NC, Lichti CF, Luisi J, Vergara F, et al. The Nav1. 2 channel is regulated by GSK3. Biochim Biophys Acta. 2015;1850(4):832–44. 25615535 10.1016/j.bbagen.2015.01.011 PMC4336163

[63] Hien YE, Montersino A, Castets F, Leterrier C, Filhol O, Vacher H, et al. CK2 accumulation at the axon initial segment depends on sodium channel Nav1. FEBS Lett. 2014;588(18):3403–8. 25109776 10.1016/j.febslet.2014.07.032

[64] Few WP, Scheuer T, Catterall WA. Dopamine modulation of neuronal Na+ channels requires binding of A kinase-anchoring protein 15and PKA by a modified leucine zipper motif. Proc Natl Acad Sci U S A. 2007;104(12):5187–92. 17360357 10.1073/pnas.0611619104 PMC1829284

[65] Beacham D, Ahn M, Catterall WA, Scheuer T. Sites and molecular mechanisms of modulation of NaV1. 2 channels by Fyn tyrosine kinase. J Neurosci. 2007;27(43):11543–51. 17959797 10.1523/JNEUROSCI.1743-07.2007 PMC6673227

[66] Wittmack EK, Rush AM, Hudmon A, Waxman SG, Dib-Hajj SD. Voltage-gated sodium channel Nav1. 6 is modulated by p38 mitogen-activated protein kinase. J Neurosci. 2005;25(28):6621–30. 16014723 10.1523/JNEUROSCI.0541-05.2005 PMC6725417

[67] Tai TY, Warner LN, Jones TD, Jung S, Concepcion FA, Skyrud DW, et al. Antiepileptic action of c-Jun N-terminal kinase (JNK) inhibition in an animal model of temporal lobe epilepsy. Neurosci. 2017;349:35–47. 10.1016/j.neuroscience.2017.02.024 PMC5386321 28237815

[68] Bréchet A, Fache MP, Brachet A, Ferracci G, Baude A, Irondelle M, et al. Protein kinase CK2 contributes to the organization of sodium channels in axonal membranes by regulating their interactions with ankyrin G. J Cell Biol. 2008;183(6):1101–14. 19064667 10.1083/jcb.200805169 PMC2600743

[69] Baek JH, Rubinstein M, Scheuer T, Trimmer JS. Reciprocal changes in phosphorylation and methylation of mammalian brain sodium channels in response to seizures. J Biol Chem. 2014;289(22):15363–73. 24737319 10.1074/jbc.M114.562785 PMC4140893

[70] Shank RP, Maryanoff BE. Molecular pharmacodynamics, clinical therapeutics, and pharmacokinetics of topiramate. CNS Neurosci Ther. 2008;14(2):120–42. 18482025 10.1111/j.1527-3458.2008.00041.x PMC6494007

[71] Cohen P. Protein kinases–the major drug targets of the twenty-first century? Nat Rev Drug Discov. 2002;1(4):309–15. 12120282 10.1038/nrd773

[72] Chico LK, Van Eldik LJ, Watterson DM. Targeting protein kinases in central nervous system disorders. Nat Rev Drug Discov. 2009;8(11):892–909. 19876042 10.1038/nrd2999 PMC2825114

[73] Hondeghem LM. Antiarrhythmic agents: modulated receptor applications. Circulation. 1987;75(3):514–20. 2434260 10.1161/01.cir.75.3.514

[74] Jensen HS, Grunnet M, Bastlund JF. Therapeutic potential of Na(V)1.1 activators. Trends Pharmacol Sci. 2014;35(3):113–8. 24439681 10.1016/j.tips.2013.12.007

[75] Adler EM, Yaari Y, David G, Selzer ME. Frequency-dependent action of phenytoin on lamprey spinal axons. Brain Res. 1986;362(2):271–80. 3942876 10.1016/0006-8993(86)90451-8

[76] Mantegazza M, Curia G, Biagini G, Ragsdale DS, Avoli M. Voltage-gated sodium channels as therapeutic targets in epilepsy and other neurological disorders. Lancet Neurol. 2010;9(4):413–24. 20298965 10.1016/S1474-4422(10)70059-4

[77] Maljevic S, Reid CA, Petrou S. Models for discovery of targeted therapy in genetic epileptic encephalopathies. J Neurochem. 2017;143(1):30–48. 28742937 10.1111/jnc.14134

[78] Cheah CS, Yu FH, Westenbroek RE, Kalume FK, Oakley JC, Potter GB, et al. Specific deletion of NaV1. 1 sodium channels in inhibitory interneurons causes seizures and premature death in a mouse model of Dravet syndrome. Proc Natl Acad Sci. 2012;109(36):14646–51. 22908258 10.1073/pnas.1211591109 PMC3437823

[79] Ogiwara I, Miyamoto H, Morita N, Atapour N, Mazaki E, Inoue I, et al. Nav1. 1 localizes to axons of parvalbumin-positive inhibitory interneurons: a circuit basis for epileptic seizures in mice carrying an Scn1a gene mutation. J Neurosci. 2007;27(22):5903–14. 17537961 10.1523/JNEUROSCI.5270-06.2007 PMC6672241

[80] Catterall WA. Sodium channels, inherited epilepsy, and antiepileptic drugs. Annu Rev Pharmacol Toxicol. 2014;54:317–38. 24392695 10.1146/annurev-pharmtox-011112-140232

[81] Ademuwagun IA, Rotimi SO, Syrbe S, Ajamma YU, Adebiyi E. Voltage gated sodium channel genes in epilepsy: mutations, functional studies, and treatment dimensions. Front Neurol. 2021;12:600050. 33841294 10.3389/fneur.2021.600050 PMC8024648

[82] Catterall WA, Kalume F, Oakley JC. NaV1.1 channels and epilepsy. J Physiol. 2010;588(Pt 11):1849–59. 20194124 10.1113/jphysiol.2010.187484 PMC2901973

[83] Suls A, Jaehn JA, Kecskés A, Weber Y, Weckhuysen S, Craiu DC, et al. De novo loss-of-function mutations in CHD2 cause a fever-sensitive myoclonic epileptic encephalopathy sharing features with Dravet syndrome. Am J Hum Genet. 2013;93(5):967–75. 24207121 10.1016/j.ajhg.2013.09.017 PMC3824114

[84] Oakley JC, Kalume F, Catterall WA. Insights into pathophysiology and therapy from a mouse model of Dravet syndrome. Epilepsia. 2011;52:59–61. 21463282 10.1111/j.1528-1167.2011.03004.x PMC3547637

[85] Wu YW, Sullivan J, McDaniel SS, Meisler MH, Walsh EM, Li SX, et al. Incidence of Dravet syndrome in a US population. Pediatrics. 2015;136(5):e1310–5. 26438699 10.1542/peds.2015-1807 PMC4621800

[86] Kaplan DI, Isom LL, Petrou S. Role of sodium channels in epilepsy. Cold Spring Harb Perspect Med. 2016;6(6):a022814. 27143702 10.1101/cshperspect.a022814 PMC4888813

[87] Armstrong CM, Bezanilla F. Currents related to movement of the gating particles of the sodium channels. Nature. 1973;242(5398):459–61. 4700900 10.1038/242459a0

[88] Nardi A, Damann N, Hertrampf T, Kless A. Advances in targeting voltage-gated sodium channels with small molecules. ChemMedChem. 2012;7(10):1712–40. 22945552 10.1002/cmdc.201200298

[89] Oakley JC, Cho AR, Cheah CS, Scheuer T, Catterall WA. Synergistic GABA-enhancing therapy against seizures in a mouse model of Dravet syndrome. J Pharmacol Exp Ther. 2013;345(2):215–24. 23424217 10.1124/jpet.113.203331 PMC3629796

[90] Kalume F, Westenbroek RE, Cheah CS, Frank HY, Oakley JC, Scheuer T, et al. Sudden unexpected death in a mouse model of Dravet syndrome. J Clin Invest. 2013;123(4):1798–808. 23524966 10.1172/JCI66220 PMC3613924

[91] Cheah CS, Westenbroek RE, Roden WH, Kalume F, Oakley JC, Jansen LA, et al. Correlations in timing of sodium channel expression, epilepsy, and sudden death in Dravet syndrome. Channels. 2013;7(6):468–72. 23965409 10.4161/chan.26023 PMC4042481

[92] Han Z, Chen C, Christiansen A, Ji S, Lin Q, Anumonwo C, et al. Antisense oligonucleotides increase Scn1a expression and reduce seizures and SUDEP incidence in a mouse model of Dravet syndrome. Sci Transl Med. 2020;12(558):eaaz6100. 32848094 10.1126/scitranslmed.aaz6100

[93] Sankar R, de Menezes MS. Metabolic and endocrine aspects of the ketogenic diet. Epilepsy Res. 1999;37(3):191–201. 10584969 10.1016/s0920-1211(99)00071-6

[94] Chiron C. Current therapeutic procedures in Dravet syndrome. Dev Med Child Neurol. 2011;53:16–8. 21504427 10.1111/j.1469-8749.2011.03967.x

[95] Jaworski T. Control of neuronal excitability by GSK-3beta: Epilepsy and beyond. Biochim Biophys Acta Mol Cell Res. 2020;1867(9):118745. 32450268 10.1016/j.bbamcr.2020.118745

[96] Cutts A, Savoie H, Hammer MF, Schreiber J, Grayson C, Luzon C, et al. Clinical characteristics and treatment experience of individuals with SCN8A developmental and epileptic encephalopathy (SCN8A-DEE): Findings from an online caregiver survey. Seizure. 2022;97:50–7. 35325842 10.1016/j.seizure.2022.03.008

[97] Møller RS, Johannesen KM. Precision medicine: SCN8A encephalopathy treated with sodium channel blockers. Neurotherapeutics. 2016;13(1):190–1. 26553437 10.1007/s13311-015-0403-5 PMC4720666

[98] Orsini A, Esposito M, Perna D, Bonuccelli A, Peroni D, Striano P. Personalized medicine in epilepsy patients. J Transl Genet Genom. 2018;2:16.

[99] Johnson JP, Focken T, Khakh K, Tari PK, Dube C, Goodchild SJ. NBI-921352, a first-in-class, NaV1. 6 selective, sodium channel inhibitor that prevents seizures in Scn8a gain-of-function mice, and wild-type mice and rats. Elife. 2022;11:e72468. 35234610 10.7554/eLife.72468 PMC8903829

[100] Bender AC, Morse RP, Scott RC, Holmes GL, Lenck-Santini PP. SCN1A mutations in Dravet syndrome: impact of interneuron dysfunction on neural networks and cognitive outcome. Epilepsy Behav. 2012;23(3):177–86. 22341965 10.1016/j.yebeh.2011.11.022 PMC3307886

[101] Scheffer IE, Nabbout R. SCN1A-related phenotypes: epilepsy and beyond. Epilepsia. 2019;60:S17-24. 31904117 10.1111/epi.16386

[102] Layer N, Sonnenberg L, Pardo González E, Benda J, Hedrich UBS, Lerche H, et al. Dravet Variant SCN1AA1783V impairs interneuron firing predominantly by altered channel activation. Front Cell Neurosci. 2021;15:754530. 34776868 10.3389/fncel.2021.754530 PMC8581729

[103] Kotler O, Khrapunsky Y, Shvartsman A, Dai H, Plant LD, Goldstein SA, et al. SUMOylation of NaV1. 2 channels regulates the velocity of backpropagating action potentials in cortical pyramidal neurons. Elife. 2023;12:e81463. 36794908 10.7554/eLife.81463 PMC10014073

[104] Spratt PW, Alexander RP, Ben-Shalom R, Sahagun A, Kyoung H, Keeshen CM, et al. Paradoxical hyperexcitability from NaV1 2 sodium channel loss in neocortical pyramidal cells. Cell Rep. 2021;36(5):109483. 34348157 10.1016/j.celrep.2021.109483 PMC8719649

[105] Hu W, Tian C, Li T, Yang M, Hou H, Shu Y. Distinct contributions of Na(v)1.6 and Na(v)1.2 in action potential initiation and backpropagation. Nat Neurosci. 2009;12(8):996–1002. 19633666 10.1038/nn.2359

[106] Schafer DP, Custer AW, Shrager P, Rasband MN. Early events in node of Ranvier formation during myelination and remyelination in the PNS. Neuron Glia Biol. 2006;2(2):69–79. 16652168 10.1017/S1740925X06000093 PMC1424668

[107] Shah BS, Stevens EB, Pinnock RD, Dixon AK, Lee K. Developmental expression of the novel voltage-gated sodium channel auxiliary subunit β3, in rat CNS. J Physiol. 2001;534(Pt 3):763–76. 11483707 10.1111/j.1469-7793.2001.t01-1-00763.x PMC2278751

[108] Misra SN, Kahlig KM, George AL Jr. Impaired NaV1. 2 function and reduced cell surface expression in benign familial neonatal-infantile seizures. Epilepsia. 2008;49(9):1535–45. 18479388 10.1111/j.1528-1167.2008.01619.x PMC3647030

[109] Sugawara T, Tsurubuchi Y, Agarwala KL, Ito M, Fukuma G, Mazaki-Miyazaki E, et al. A missense mutation of the Na+ channel alpha II subunit gene Na(v)1.2 in a patient with febrile and afebrile seizures causes channel dysfunction. Proc Natl Acad Sci U S A. 2001;98(11):6384–9. 11371648 10.1073/pnas.111065098 PMC33477

[110] Scalmani P, Rusconi R, Armatura E, Zara F, Avanzini G, Franceschetti S, et al. Effects in neocortical neurons of mutations of the Na(v)1.2 Na+ channel causing benign familial neonatal-infantile seizures. J Neurosci. 2006;26(40):10100–9. 17021166 10.1523/JNEUROSCI.2476-06.2006 PMC6674637

[111] Que Z, Olivero-Acosta MI, Zhang J, Eaton M, Tukker AM, Chen X, et al. Hyperexcitability and pharmacological responsiveness of cortical neurons derived from human iPSCs carrying epilepsy-associated sodium channel Nav 1. 2–L1342P genetic variant. J Neurosci. 2021;41(49):10194–208. 34716231 10.1523/JNEUROSCI.0564-21.2021 PMC8660047

[112] Zavala-Tecuapetla C, Manjarrez-Marmolejo J, Ramírez-Jarquín JO, Rivera-Cerecedo CV. Eslicarbazepine, but not lamotrigine or ranolazine, shows anticonvulsant efficacy in carbamazepine-resistant rats developed by window-pentylenetetrazole kindling. Brain Sci. 2022;12(5):629. 35625015 10.3390/brainsci12050629 PMC9139658

[113] Vacher H, Mohapatra DP, Trimmer JS. Localization and targeting of voltage-dependent ion channels in mammalian central neurons. Physiol Rev. 2008;88(4):1407–47. 18923186 10.1152/physrev.00002.2008 PMC2587220

[114] Hill AS, Nishino A, Nakajo K, Zhang G, Fineman JR, Selzer ME, et al. Ion channel clustering at the axon initial segment and node of Ranvier evolved sequentially in early chordates. PLoS Genet. 2008;4(12):e1000317. 19112491 10.1371/journal.pgen.1000317 PMC2597720

[115] Menezes LF, Sabiá Júnior EF, Tibery DV, Carneiro LD, Schwartz EF. Epilepsy-related voltage-gated sodium channelopathies: a review. Front Pharmacol. 2020;11:1276. 33013363 10.3389/fphar.2020.01276 PMC7461817

[116] Blumenfeld H, Lampert A, Klein JP, Mission J, Chen MC, Rivera M, et al. Role of hippocampal sodium channel Nav1. 6 in kindling epileptogenesis. Epilepsia. 2009;50(1):44–55. 18637833 10.1111/j.1528-1167.2008.01710.x PMC3741044

[117] Hargus NJ, Nigam A, Bertram EH III, Patel MK. Evidence for a role of Nav1. 6 in facilitating increases in neuronal hyperexcitability during epileptogenesis. J Neurophysiol. 2013;110(5):1144–57. 23741036 10.1152/jn.00383.2013 PMC3763090

[118] Johannesen KM, Gardella E, Scheffer I, Howell K, Smith DM, Helbig I, et al. Early mortality in SCN8A-related epilepsies. Epilepsy Res. 2018;143:79–81. 29677576 10.1016/j.eplepsyres.2018.04.008

[119] Wang J, Gao H, Bao X, Zhang Q, Li J, Wei L, et al. SCN8A mutations in Chinese patients with early onset epileptic encephalopathy and benign infantile seizures. BMC Med Genet. 2017;18(1):1. 28923014 10.1186/s12881-017-0460-1 PMC5604297

[120] Meisler MH, Helman G, Hammer MF, Fureman BE, Gaillard WD, Goldin AL, et al. SCN8A encephalopathy: research progress and prospects. Epilepsia. 2016;57(7):1027–35. 27270488 10.1111/epi.13422 PMC5495462

[121] Arafat A, Jing P, Ma Y, Pu M, Nan G, Fang H, et al. Unexplained early infantile epileptic encephalopathy in Han Chinese children: next-generation sequencing and phenotype enriching. Sci Rep. 2017;7(1):46227. 28387369 10.1038/srep46227 PMC5384237

[122] Mastrangelo M, Leuzzi V. Genes of early-onset epileptic encephalopathies: from genotype to phenotype. Pediatr Neurol. 2012;46(1):24–31. 22196487 10.1016/j.pediatrneurol.2011.11.003

[123] Gardella E, Marini C, Trivisano M, Fitzgerald MP, Alber M, Howell KB, Darra F, Siliquini S, Bölsterli BK, Masnada S, Pichiecchio A. The phenotype of SCN8A developmental and epileptic encephalopathy. Neurology. 2018;91(12):e1112–24. 30171078 10.1212/WNL.0000000000006199

[124] Blair RD. Temporal lobe epilepsy semiology. Epilepsy Res Treat. 2012;2012:751510. 22957241 10.1155/2012/751510 PMC3420439

[125] Whitaker WR, Faull RL, Dragunow M, Mee EW, Emson PC, Clare JJ. Changes in the mRNAs encoding voltage-gated sodium channel types II and III in human epileptic hippocampus. Neuroscience. 2001;106(2):275–85. 11566500 10.1016/s0306-4522(01)00212-3

[126] Vreugdenhil M, Hoogland G, Van Veelen CW, Wadman WJ. Persistent sodium current in subicular neurons isolated from patients with temporal lobe epilepsy. Eur J Neurosci. 2004;19(10):2769–78. 15147310 10.1111/j.1460-9568.2004.03400.x

[127] Rosker C, Lohberger B, Hofer D, Steinecker B, Quasthoff S, Schreibmayer W. The TTX metabolite 4,9-anhydro-TTX is a highly specific blocker of the Na(v1.6) voltage-dependent sodium channel. Am J Physiol Cell Physiol. 2007;293(2):C783-9. 17522141 10.1152/ajpcell.00070.2007

[128] Hargus NJ, Merrick EC, Nigam A, Kalmar CL, Baheti AR, Bertram EH III, et al. Temporal lobe epilepsy induces intrinsic alterations in Na channel gating in layer II medial entorhinal cortex neurons. Neurobiol Dis. 2011;41(2):361–76. 20946956 10.1016/j.nbd.2010.10.004 PMC3014455

[129] Wagnon JL, Meisler MH. Recurrent and non-recurrent mutations of SCN8A in epileptic encephalopathy. Front Neurol. 2015;6:104. 26029160 10.3389/fneur.2015.00104 PMC4432670

[130] Maguire MJ, Jackson CF, Marson AG, Nevitt SJ. Treatments for the prevention of Sudden Unexpected Death in Epilepsy (SUDEP). Cochrane Database Syst Rev. 2016;7(7):CD011792. 32239759 10.1002/14651858.CD011792.pub3 PMC7115126

[131] Larsen J, Carvill GL, Gardella E, Kluger G, Schmiedel G, Barisic N, et al. The phenotypic spectrum of SCN8A encephalopathy. Neurology. 2015;84(5):480–9. 25568300 10.1212/WNL.0000000000001211 PMC4336074

[132] Boerma RS, Braun KP, van de Broek MP, van Berkestijn FM, Swinkels ME, Hagebeuk EO, et al. Remarkable phenytoin sensitivity in 4 children with SCN8A-related epilepsy: a molecular neuropharmacological approach. Neurotherapeutics. 2016;13:192–7. 26252990 10.1007/s13311-015-0372-8 PMC4720675

[133] Kuo CC, Bean BP. Slow binding of phenytoin to inactivated sodium channels in rat hippocampal neurons. Mol Pharmacol. 1994;46(4):716–25. 7969051

[134] Barker BS, Ottolini M, Wagnon JL, Hollander RM, Meisler MH, Patel MK. The SCN8A encephalopathy mutation p. Ile1327Val displays elevated sensitivity to the anticonvulsant phenytoin. Epilepsia. 2016;57(9):1458–66. 27375106 10.1111/epi.13461 PMC5012949

[135] Braakman HM, Verhoeven JS, Erasmus CE, Haaxma CA, Willemsen MH, Schelhaas HJ. Phenytoin as a last-resort treatment in SCN 8A encephalopathy. Epilepsia Open. 2017;2(3):343–4. 29588963 10.1002/epi4.12059 PMC5862112

[136] Ko A, Kang HC. Frequently identified genetic developmental and epileptic encephalopathy: a review focusing on precision medicine. Ann Child Neurol. 2019;27(1):2–12.

[137] Löscher W, Klein P. The pharmacology and clinical efficacy of antiseizure medications: from bromide salts to cenobamate and beyond. CNS Drugs. 2021;35(9):935–63. 34145528 10.1007/s40263-021-00827-8 PMC8408078

[138] Fattorusso A, Matricardi S, Mencaroni E, Dell’Isola GB, Di Cara G, Striano P, et al. The pharmacoresistant epilepsy: an overview on existant and new emerging therapies. Front Neurol. 2021;12:674483. 34239494 10.3389/fneur.2021.674483 PMC8258148

[139] Androsova G, Krause R, Borghei M, Wassenaar M, Auce P, Avbersek A, et al. Comparative effectiveness of antiepileptic drugs in patients with mesial temporal lobe epilepsy with hippocampal sclerosis. Epilepsia. 2017;58(10):1734–41. 28857179 10.1111/epi.13871

[140] Vreugdenhil M, Wadman WJ. Modulation of sodium currents in rat CA1 neurons by carbamazepine and valproate after kindling epileptogenesis. Epilepsia. 1999;40(11):1512–22. 10565577 10.1111/j.1528-1157.1999.tb02034.x

[141] Remy S, Gabriel S, Urban BW, Dietrich D, Lehmann TN, Elger CE, et al. A novel mechanism underlying drug resistance in chronic epilepsy. Ann Neurol. 2003;53(4):469–79. 12666114 10.1002/ana.10473

[142] Schaub C, Uebachs M, Beck H. Diminished response of CA1 neurons to antiepileptic drugs in chronic epilepsy. Epilepsia. 2007;48(7):1339–50. 17441992 10.1111/j.1528-1167.2007.01103.x

[143] Remy S, Urban BW, Elger CE, Beck H. Anticonvulsant pharmacology of voltage-gated Na+ channels in hippocampal neurons of control and chronically epileptic rats. Eur J Neurosci. 2003;17(12):2648–58. 12823472 10.1046/j.1460-9568.2003.02710.x

[144] Jeub M, Beck H, Siep E, Rüschenschmidt C, Speckmann EJ, Ebert U. Effect of phenytoin on sodium and calcium currents in hippocampal CA1 neurons of phenytoin-resistant kindled rats. Neuropharmacology. 2002;42(1):107–16. 11750920 10.1016/s0028-3908(01)00148-4

[145] Alexopoulos AV. Pharmacoresistant epilepsy: definition and explanation. Epileptology. 2013;1(1):38–42.

[146] Sourbron J, Auvin S, Arzimanoglou A, Cross JH, Hartmann H, Pressler R, et al. Medical treatment in infants and young children with epilepsy: Off-label use of antiseizure medications. Survey Report of ILAE Task Force Medical Therapies in Children. Epilepsia Open. 2023;8(1):77–89. 36281833 10.1002/epi4.12666 PMC9977757

[147] Asadi-Pooya AA, Beniczky S, Rubboli G, Sperling MR, Rampp S, Perucca E. A pragmatic algorithm to select appropriate antiseizure medications in patients with epilepsy. Epilepsia. 2020;61(8):1668–77. 32697354 10.1111/epi.16610

[148] Nolan SJ, Tudur Smith C, Weston J, Marson AG. Lamotrigine versus carbamazepine monotherapy for epilepsy: an individual participant data review. Cochrane Database Syst Rev. 2016;11(11):CD001031. 27841445 10.1002/14651858.CD001031.pub3 PMC6478073

[149] Gambardella A, Labate A, Colosimo E, Ambrosio R, Quattrone A. Monotherapy for partial epilepsy: focus on levetiracetam. Neuropsychiatr Dis Treat. 2008;4(1):33–8. 18728811 10.2147/ndt.s1655 PMC2515905

[150] Aras LM, Isla J, Mingorance-Le MA. The European patient with Dravet syndrome: results from a parent-reported survey on antiepileptic drug use in the European population with Dravet syndrome. Epilepsy Behav. 2015;44:104–9. 25666511 10.1016/j.yebeh.2014.12.028

[151] Barker-Haliski M, White HS. Validated animal models for antiseizure drug (ASD) discovery: advantages and potential pitfalls in ASD screening. Neuropharmacology. 2020;167:107750. 31469995 10.1016/j.neuropharm.2019.107750 PMC7470169

[152] Santulli L, Coppola A, Balestrini S, Striano S. The challenges of treating epilepsy with 25 antiepileptic drugs. Pharmacol Res. 2016;107:211–9. 26995307 10.1016/j.phrs.2016.03.016

[153] Gaily E, Anttonen AK, Valanne L, Liukkonen E, Träskelin AL, Polvi A, et al. Dravet syndrome: new potential genetic modifiers, imaging abnormalities, and ictal findings. Epilepsia. 2013;54(9):1577–85. 23808377 10.1111/epi.12256

[154] Dressler A, Trimmel-Schwahofer P, Reithofer E, Mühlebner A, Gröppel G, Reiter-Fink E, et al. Efficacy and tolerability of the ketogenic diet in Dravet syndrome–comparison with various standard antiepileptic drug regimen. Epilepsy Res. 2015;109:81–9. 25524846 10.1016/j.eplepsyres.2014.10.014

[155] Inoue Y, Ohtsuka Y, Study Group. Long-term safety and efficacy of stiripentol for the treatment of Dravet syndrome: a multicenter, open-label study in Japan. Epilepsy Res. 2015;113:90–7. 25986195 10.1016/j.eplepsyres.2015.03.012

[156] Xu C, Zhang Y, Gozal D, Carney P. Channelopathy of Dravet syndrome and potential neuroprotective effects of cannabidiol. J Cent Nerv Syst Dis. 2021;13:11795735211048044. 34992485 10.1177/11795735211048045 PMC8724990

[157] Amrutkar C, Riel-Romero RM. Lennox Gastaut Syndrome. In: StatPearls. Treasure Island: StatPearls Publishing; 2023. 30422560

[158] Auvin S, Damera V, Martin M, Holland R, Simontacchi K, Saich A. The impact of seizure frequency on quality of life in patients with Lennox-Gastaut syndrome or Dravet syndrome. Epilepsy Behav. 2021;123:108239. 34375802 10.1016/j.yebeh.2021.108239

[159] Abu Saleh T, Stephen L. Lennox gastaut syndrome, review of the literature and a case report. Head Face Med. 2008;4:1–7. 18541034 10.1186/1746-160X-4-9 PMC2483705

[160] Camfield PR. Definition and natural history of Lennox-Gastaut syndrome. Epilepsia. 2011;52:3–9. 21790560 10.1111/j.1528-1167.2011.03177.x

[161] Nordli RD. Epileptic encephalopathies in infancy and early childhood: Overview. Atlas of Epilepsies. Oxford: Springer-Verlag London Limited; 2010. p. 881–3.

[162] Romoli M, Mazzocchetti P, D’Alonzo R, Siliquini S, Rinaldi VE, Verrotti A, et al. Valproic acid and epilepsy: from molecular mechanisms to clinical evidences. Curr Neuropharmacol. 2019;17(10):926–46. 30592252 10.2174/1570159X17666181227165722 PMC7052829

[163] Sazgar M, Bourgeois BF. Aggravation of epilepsy by antiepileptic drugs. Pediatr Neurol. 2005;33(4):227–34. 16194719 10.1016/j.pediatrneurol.2005.03.001

[164] Devi N, Madaan P, Ameen R, Sahu JK, Bansal D. Short-term and long-term efficacy and safety of antiseizure medications in Lennox Gastaut syndrome: a network meta-analysis. Seizure. 2022;99:164–75. 35487871 10.1016/j.seizure.2022.04.004

[165] Guerreiro MM, Manreza ML, Scotoni AE, Silva EA, Guerreiro CA, Souza EA, et al. A pilot study of topiramate in children with Lennox-Gastaut syndrome. Arq Neuropsiquiatr. 1999;57(2A):167–75. 10412513 10.1590/s0004-282x1999000200001

[166] Coppola G, Caliendo G, Veggiotti P, Romeo A, Tortorella G, De Marco P, et al. Topiramate as add-on drug in children, adolescents and young adults with Lennox-Gastaut syndrome: an Italian multicentric study. Epilepsy Res. 2002;51(1–2):147–53. 12350390 10.1016/s0920-1211(02)00103-1

[167] Tartara A, Sartori I, Manni R, Galimberti CA, Di Fazio M, Perucca E. Efficacy and safety of topiramate in refractory epilepsy: a long-term prospective trial. Ital J Neurol Sci. 1996;17:429–32. 8978450 10.1007/BF01997718

[168] Grosso S, Franzoni E, Iannetti P, Incorpora G, Cardinali C, Toldo I, et al. Efficacy and safety of topiramate in refractory epilepsy of childhood: long-term follow-up study. J Child Neurol. 2005;20(11):893–7. 16417859 10.1177/08830738050200110601

[169] Conradsen I, Wolf P, Sams T, Sorensen HB, Beniczky S. Patterns of muscle activation during generalized tonic and tonic–clonic epileptic seizures. Epilepsia. 2011;52(11):2125–32. 21973264 10.1111/j.1528-1167.2011.03286.x

[170] Langtry HD, Gillis JC, Davis R. Topiramate: a review of its pharmacodynamic and pharmacokinetic properties and clinical efficacy in the management of epilepsy. Drugs. 1997;54:752–73. 9360061 10.2165/00003495-199754050-00009

[171] Balagura G, Riva A, Marchese F, Verrotti A, Striano P. Adjunctive rufinamide in children with Lennox-Gastaut syndrome: a literature review. Neuropsychiatr Dis Treat. 2020;16:369–79. 32103957 10.2147/NDT.S185774 PMC7008198

[172] Kluger G, Bauer B. Role of rufinamide in the management of Lennox-Gastaut syndrome (childhood epileptic encephalopathy). Neuropsychiatr Dis Treat. 2007;3(1):3–11. 19300535 10.2147/nedt.2007.3.1.3 PMC2654531

[173] Kim SH, Eun SH, Kang HC, Kwon EJ, Byeon JH, Lee YM, et al. Rufinamide as an adjuvant treatment in children with Lennox-Gastaut syndrome. Seizure. 2012;21(4):288–91. 22421185 10.1016/j.seizure.2012.02.006

[174] Coppola G, Grosso S, Franzoni E, Veggiotti P, Zamponi N, Parisi P, et al. Rufinamide in children and adults with Lennox-Gastaut syndrome: first Italian multicenter experience. Seizure. 2010;19(9):587–91. 20888268 10.1016/j.seizure.2010.09.008

[175] You SJ, Kang HC, Kim HD, Lee HS, Ko TS. Clinical efficacy of zonisamide in Lennox-Gastaut syndrome: Korean multicentric experience. Brain Dev. 2008;30(4):287–90. 17959327 10.1016/j.braindev.2007.09.004

[176] Cross JH, Auvin S, Falip M, Striano P, Arzimanoglou A. Expert opinion on the management of Lennox-Gastaut syndrome: treatment algorithms and practical considerations. Front Neurol. 2017;8:505. 29085326 10.3389/fneur.2017.00505 PMC5649136

[177] Yamauchi T, Aikawa H. Efficacy of zonisamide: our experience. Seizure. 2004;13:S41–8. 15511689 10.1016/j.seizure.2004.04.021

[178] Karimzadeh P, Ashrafi MR, Bali MK, Nasehi MM, Otaghsara SM, Taghdiri MM, et al. Zonisamide efficacy as adjunctive therapy in children with refractory epilepsy. Iran J Child Neurol. 2013;7(2):37. 24665295 PMC3943038

[179] Motte J, Trevathan E, Arvidsson JF, Barrera MN, Mullens EL, Manasco P, et al. Lamotrigine for generalized seizures associated with the Lennox-Gastaut syndrome. N Engl J Med. 1997;337(25):1807–12. 9400037 10.1056/NEJM199712183372504

[180] Schapel GJ, Beran RG, Vajda FJ, Berkovic SF, Mashford ML, Dunagan FM, et al. Double-blind, placebo controlled, crossover study of lamotrigine in treatment resistant partial seizures. J Neurol Neurosurg Psychiatry. 1993;56(5):448–53. 8505632 10.1136/jnnp.56.5.448 PMC1014998

[181] Buchanan N. The efficacy of lamotrigine on seizure control in 34 children, adolescents and young adults with intellectual and physical disability. Seizure. 1995;4(3):233–6. 7582659 10.1016/s1059-1311(05)80066-4

[182] Marciani MG, Spanedda F, Bassetti MA, Maschio M, Gigli GL, Mattia D, et al. Effect of lamotrigine on EEG paroxysmal abnormalities and background activity: a computerized analysis. Br J Clin Pharmacol. 1996;42(5):621–7. 8951194 10.1111/j.1365-2125.1996.tb00057.x

[183] Boas J, Dam M, Friis ML, Kristensen O, Pedersen B, Gallagher J. Controlled trial of lamotrigine (Lamictal®) for treatment-resistant partial seizures. Acta Neurol Scand. 1996;94(4):247–52. 8937535 10.1111/j.1600-0404.1996.tb07060.x

[184] Schmidt D, Bourgeois B. A risk-benefit assessment of therapies for Lennox-Gastaut syndrome. Drug Saf. 2000;22(6):467–77. 10877040 10.2165/00002018-200022060-00005

[185] Saitsu H, Kato M, Okada I, Orii KE, Higuchi T, Hoshino H, et al. STXBP1 mutations in early infantile epileptic encephalopathy with suppression-burst pattern. Epilepsia. 2010;51(12):2397–405. 20887364 10.1111/j.1528-1167.2010.02728.x

[186] Wu MJ, Hu CH, Ma JH, Hu JS, Liu ZS, Sun D. Early infantile epileptic encephalopathy caused by PACS2 gene variation: three cases report and literature review. Zhonghua Er Ke Za Zhi. 2021;59(7):594–9 (Chinese). 34405643 10.3760/cma.j.cn112140-20201122-01047

[187] Zhong M, Liao S, Li T, Wu P, Wang Y, Wu F, et al. Early diagnosis improving the outcome of an infant with epileptic encephalopathy with cytoplasmic FMRP interacting protein 2 mutation: case report and literature review. Medicine(Baltimore). 2019;98(44):e17749. 31689829 10.1097/MD.0000000000017749 PMC7017979

[188] Dilena R, Striano P, Gennaro E, Bassi L, Olivotto S, Tadini L, et al. Efficacy of sodium channel blockers in SCN2A early infantile epileptic encephalopathy. Brain Dev. 2017;39(4):345–8. 27876397 10.1016/j.braindev.2016.10.015

[189] Guberman A. Monotherapy or polytherapy for epilepsy? Can J Neurol Sci. 1998;25(S4):S3-8. 9827238 10.1017/s0317167100034892

[190] Morris JC, Dodson WE, Hatlelid JM, Ferrendelli JA. Phenytoin and carbamazepine, alone and in combination: anticonvulsant and neurotoxic effects. Neurology. 1987;37(7):1111. 3601077 10.1212/wnl.37.7.1111

[191] Sperling MR, Harvey J, Grinnell T, Cheng H, Blum D, 045 Study Team. Efficacy and safety of conversion to monotherapy with eslicarbazepine acetate in adults with uncontrolled partial-onset seizures: a randomized historical-control phase III study based in North America. Epilepsia. 2015;56(4):546–55. 25689448 10.1111/epi.12934 PMC5016771

[192] Jacobson MP, Pazdera L, Bhatia P, Grinnell T, Cheng H, Blum D, et al. Efficacy and safety of conversion to monotherapy with eslicarbazepine acetate in adults with uncontrolled partial-onset seizures: a historical-control phase III study. BMC Neurol. 2015;15:46. 25880756 10.1186/s12883-015-0305-5 PMC4397697

[193] Sperling MR, French J, Jacobson MP, Pazdera L, Gough M, Cheng H, et al. Conversion to eslicarbazepine acetate monotherapy: a pooled analysis of 2 phase III studies. Neurology. 2016;86(12):1095–102. 26911639 10.1212/WNL.0000000000002497 PMC4826334

[194] Krauss G, Biton V, Harvey JH, Elger C, Trinka E, da Silva PS, et al. Influence of titration schedule and maintenance dose on the tolerability of adjunctive eslicarbazepine acetate: an integrated analysis of three randomized placebo-controlled trials. Epilepsy Res. 2018;139:1–8. 29127848 10.1016/j.eplepsyres.2017.10.021

[195] Jallon P, Picard F. Bodyweight gain and anticonvulsants: a comparative review. Drug Saf. 2001;24:969–78. 11735653 10.2165/00002018-200124130-00004

[196] Kwan P, Brodie MJ. Drug treatment of epilepsy: when does it fail and how to optimize its use? CNS Spectr. 2004;9(2):110–9. 14999167 10.1017/s1092852900008476

[197] Mattson RH, Cramer JA, Collins JF, Smith DB, Delgado-Escueta AV, Browne TR, et al. Comparison of carbamazepine, phenobarbital, phenytoin, and primidone in partial and secondarily generalized tonic–clonic seizures. N Engl J Med. 1985;313(3):145–51. 3925335 10.1056/NEJM198507183130303

[198] Lee JW, Dworetzky B. Rational polytherapy with antiepileptic drugs. Pharmaceuticals. 2010;3(8):2362–79. 27713357 10.3390/ph3082362 PMC4033928

[199] St Louis EK. Truly “rational” polytherapy: maximizing efficacy and minimizing drug interactions, drug load, and adverse effects. Curr Neuropharmacol. 2009;7(2):96–105. 19949567 10.2174/157015909788848929 PMC2730011

[200] Deckers CL, Hekster YA, Keyser A, Van Lier HJ, Meinardi H, Renier WO. Monotherapy versus polytherapy for epilepsy: a multicenter double-blind randomized study. Epilepsia. 2001;42(11):1387–94. 11879339 10.1046/j.1528-1157.2001.30800.x

[201] Reynolds EH, Shorvon SD. Monotherapy or poly therapy for epilepsy? Epilepsia. 1981;22(1):1. 6781884 10.1111/j.1528-1157.1981.tb04327.x

[202] Czuczwar SJ, Borowicz KK. Polytherapy in epilepsy: the experimental evidence. Epilepsy Res. 2002;52(1):15–23. 12445956 10.1016/s0920-1211(02)00181-x

[203] Armour DJ, Veitch GB. Is valproate monotherapy a practical possibility in chronically uncontrolled epilepsy? J Clin Pharm Ther. 1988;13(1):53–64. 3129441 10.1111/j.1365-2710.1988.tb00506.x

[204] Harden CL, Zisfein J, Atos-Radzion EC, Tuchman AJ. Combination valproate—carbamazepine therapy in partial epilepsies resistant to carbamazepine monotherapy. J Epilepsy. 1993;6(2):91–4.

[205] Mj B. Yuen AWC and the 105-study group lamotrigine substitution study: evidence for synergism with sodium valproate. Epilepsy Res. 1997;26:423–32. 9127723 10.1016/s0920-1211(96)01007-8

[206] Stephen LJ, Sills GJ, Brodie MJ. Lamotrigine and topiramate may be a useful combination. Lancet. 1998;351(9107):958–9. 9734949 10.1016/S0140-6736(05)60613-7

[207] Read CL, Stephen LJ, Stolarek IH, Paul A, Sills GJ, Brodie MJ. Cognitive effects of anticonvulsant monotherapy in elderly patients: a placebo-controlled study. Seizure. 1998;7(2):159–62. 9627208 10.1016/s1059-1311(98)80073-3

[208] Brodie MJ. Pharmacological treatment of drug-resistant epilepsy in adults: a practical guide. Curr Neurol Neurosci Rep. 2016;16(9):82. 27443649 10.1007/s11910-016-0678-x

[209] Chang XC, Yuan H, Wang Y, Xu HQ, Hong WK, Zheng RY. Eslicarbazepine acetate add-on therapy for drug-resistant focal epilepsy. Cochrane Database Syst Rev. 2021;6(6):CD008907. 34155624 10.1002/14651858.CD008907.pub4 PMC8218144

[210] Al‐Bachari S, Pulman J, Hutton JL, Marson AG. Gabapentin add‐on for drug‐resistant partial epilepsy. Cochrane Database Syst Rev. 2013(7):CD001415. 10.1002/14651858.CD001415.pub2 23888424

[211] Pulman J, Jette N, Dykeman J, Hemming K, Hutton JL, Marson AG. Topiramate add-on for drug-resistant partial epilepsy. Cochrane Database Syst Rev. 2014;2:CD001417. 10.1002/14651858.CD001417.pub3 24570033

[212] Levisohn PM. Safety and tolerability of topiramate in children. J Child Neurol. 2000;15(Suppl 1):S22–6. 11218053 10.1177/0883073800015001S05

[213] Angehagen M, Rönnbäck L, Hansson E, Ben-Menachem E. Topiramate reduces AMPA-induced Ca(2+) transients and inhibits GluR1 subunit phosphorylation in astrocytes from primary cultures. J Neurochem. 2005;94(4):1124–30. 16092949 10.1111/j.1471-4159.2005.03259.x

[214] Korinthenberg R, Schreiner A. Topiramate in children with west syndrome: a retrospective multicenter evaluation of 100 patients. J Child Neurol. 2007;22(3):302–6. 17621500 10.1177/0883073807300535

[215] van Rijckevorsel K. Treatment of Lennox-Gastaut syndrome: overview and recent findings. Neuropsychiatr Dis Treat. 2008;4(6):1001–19. 19337447 10.2147/ndt.s1668 PMC2646636

[216] Verrotti A, Striano P, Iapadre G, Zagaroli L, Bonanni P, Coppola G, et al. The pharmacological management of Lennox-Gastaut syndrome and critical literature review. Seizure. 2018;63:17–25. 30391662 10.1016/j.seizure.2018.10.016

[217] Kwan P, Brodie MJ. Combination therapy in epilepsy: when and what to use. Drugs. 2006;66:1817–29. 17040113 10.2165/00003495-200666140-00004

[218] Canevini MP, De Sarro G, Galimberti CA, Gatti G, Licchetta L, Malerba A, et al. Relationship between adverse effects of antiepileptic drugs, number of coprescribed drugs, and drug load in a large cohort of consecutive patients with drug-refractory epilepsy. Epilepsia. 2010;51(5):797–804. 20545754 10.1111/j.1528-1167.2010.02520.x

[219] Lammers MW, Hekster YA, Keyser A, Meinardi H, Renier WO, Van Lier H. Monotherapy or polytherapy for epilepsy revisited: a quantitative assessment. Epilepsia. 1995;36(5):440–6. 7614920 10.1111/j.1528-1157.1995.tb00484.x

