# Correction: Therapeutic efficacy of voltage-gated sodium channel inhibitors in epilepsy

**DOI:** 10.1186/s42494-024-00196-x

**Published:** 2024-12-04

**Authors:** John Agbo, Zainab G. Ibrahim, Shehu Y. Magaji, Yahkub Babatunde Mutalub, Philemon Paul Mshelia, Daniel H. Mhya

**Affiliations:** 1 Department of Clinical Pharmacology and Therapeutics, Faculty of Basic Clinical Sciences, College of Medical Sciences, Abubakar Tafawa Balewa University, Bauchi, 740,272 Nigeria; 2 Department of Physiology, Faculty of Basic Medical Science, College of Medical Sciences, Abubakar Tafawa Balewa University, Bauchi, 740,272 Nigeria; 3 Department of Medical Biochemistry, Faculty of Basic Medical Science, College of Medical Sciences, Abubakar Tafawa Balewa University, Bauchi, 740,272 Nigeria

**Correction: Acta Epileptologica 5, 16 (2023)**

**https://doi.org/10.1186/s42494-023–00127-2**

Following publication of the original article [1], the authors reported an error in the author’s name, where Daniel H. Mhyha should be corrected to Daniel H. Mhya.

The original article [1] has been updated.
